# A partial form of AIRE deficiency underlies a mild form of autoimmune polyendocrine syndrome type 1

**DOI:** 10.1172/JCI169704

**Published:** 2023-11-01

**Authors:** Bergithe Eikeland Oftedal, Amund Holte Berger, Øyvind Bruserud, Yael Goldfarb, Andre Sulen, Lars Breivik, Alexander Hellesen, Shifra Ben-Dor, Rebecca Haffner-Krausz, Per M. Knappskog, Stefan Johansson, Anette S.B. Wolff, Eirik Bratland, Jakub Abramson, Eystein Sverre Husebye

**Affiliations:** 1 Department of Clinical Science and KG Jebsen Center for Autoimmune Diseases, University of Bergen, Norway.; 2 Department of Medicine and; 3 Department of Medical Genetics, Haukeland University Hospital, Bergen, Norway.; 4 Department of Immunology and Regenerative Biology, Weizmann Institute of Science, Rehovot, Israel.; 5 Bioinformatics Unit, Department of Life Sciences Core Facilities and; 6 Department of Veterinary Resources, Weizmann Institute of Science, Rehovot, Israel.

**Keywords:** Autoimmunity, Endocrinology, Adaptive immunity, Genetic variation, Tolerance

## Abstract

Autoimmune polyendocrine syndrome type 1 (APS-1) is caused by mutations in the autoimmune regulator (*AIRE*) gene. Most patients present with severe chronic mucocutaneous candidiasis and organ-specific autoimmunity from early childhood, but the clinical picture is highly variable. AIRE is crucial for negative selection of T cells, and scrutiny of different patient mutations has previously highlighted many of its molecular mechanisms. In patients with a milder adult-onset phenotype sharing a mutation in the canonical donor splice site of intron 7 (c.879+1G>A), both the predicted altered splicing pattern with loss of exon 7 (Aire^Ex7–/–^) and normal full-length *AIRE* mRNA were found, indicating leaky rather than abolished mRNA splicing. Analysis of a corresponding mouse model demonstrated that the Aire^Ex7–/–^ mutant had dramatically impaired transcriptional capacity of tissue-specific antigens in medullary thymic epithelial cells but still retained some ability to induce gene expression compared with the complete loss-of-function Aire^C313X–/–^ mutant. Our data illustrate an association between AIRE activity and the severity of autoimmune disease, with implications for more common autoimmune diseases associated with AIRE variants, such as primary adrenal insufficiency, pernicious anemia, type 1 diabetes, and rheumatoid arthritis.

## Graphical abstract

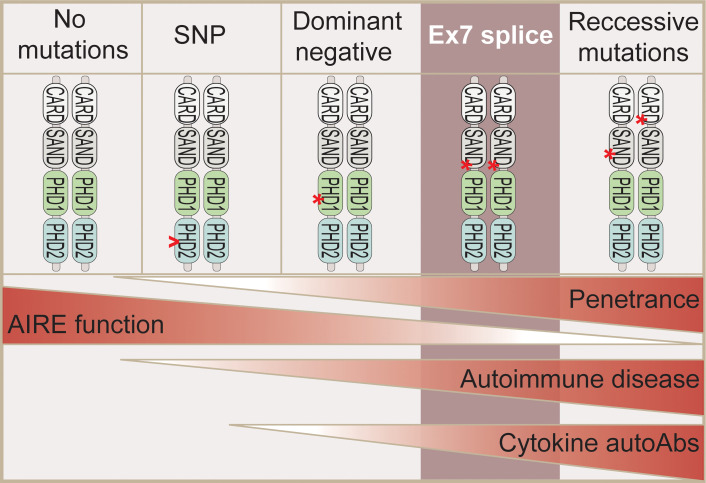

## Introduction

Autoimmune polyendocrine syndrome type 1 (APS-1) or autoimmune polyendocrinopathy-candidiasis-ectodermal dystrophy (APECED) (OMIM #240300) is a rare, childhood-onset disorder caused by mutations in the autoimmune regulator (*AIRE)* gene (1–4). It is clinically defined by the presence of 2 of the 3 major disease components: primary adrenal insufficiency, hypoparathyroidism, and chronic mucocutaneous candidiasis (1, 5), but the clinical phenotype also includes several minor disease components (6, 7). *AIRE* is almost exclusively expressed in the thymus (8), where it functions as a transcriptional regulator, crucial for negative selection of self-reactive T cells and development of Tregs (9, 10).

The human *AIRE* gene contains 14 exons that encode a protein with 545 amino acids and a has molecular weight of 57.5 kDa (3, 4). To date, over 100 mutations in the *AIRE* gene have been reported, with several predicted to affect mRNA splicing. This is a process involving large complexes of protein and RNA that catalyze the removal of introns from nuclear pre-mRNA called spliceosomes (11). Mutations occurring within splice sites can alter or abolish splicing or change the balance of alternative mRNA splice forms, leading to structural and functional changes in the protein (12). Information on the functional characterization and clinical correlations of splice mutations the *AIRE* gene is sparse (13–15).

We scrutinized the Norwegian APS-1 cohort and identified 4 patients from 3 families from a small region in Norway, who shared a disease picture characterized by a conspicuously late disease onset and extended time intervals between new disease components. They were either homozygous (3) or compound heterozygous (1) for the same *AIRE* mutation, a c.879+1G>A variant. This variant is located in the canonical splice donor site of intron 7 and predicted to cause skipping of exon 7 and loss of the linking region between the SAND and PHD1 domains (16). Functional studies in vitro and in a corresponding mouse model support a clear relationship between AIRE function and the degree of autoimmunity, which explains the milder phenotype.

## Results

### Later disease onset and milder phenotype in APS-1 patients with the c.879+1G>A AIRE mutation.

**Figure 1:**
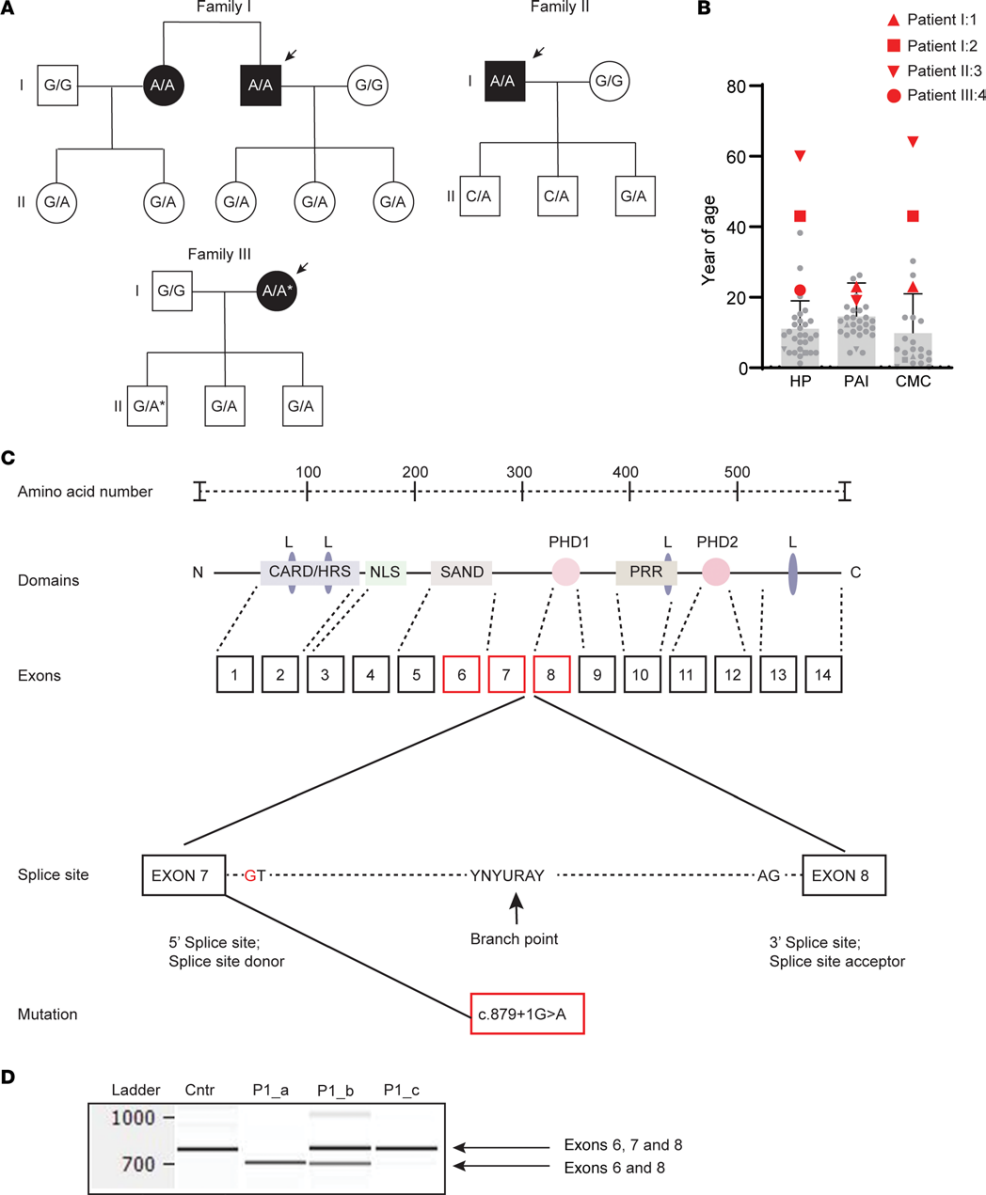
Pedigree of the 3 families studied and schematic representation of the AIRE protein, the *AIRE* gene, and the splice mutation c.879+1G>A. (**A**) Overview of the 3 families studied. All index patients have children who are carriers for monoallelic disease-causing *AIRE* mutations without any clinical disease manifestations or typical APS-1 autoantibodies. Genotypes are shown as the allelic combinations of WT (G), c.879+1G>A (A), and c.967_979del13 (A*). The arrows indicate the patients with APS-1. (**B**) The patients (red data points) harboring the c.879+1G>A had a later debut age for the 3 main manifestations of hypoparathyroidism (HP), autoimmune Addison’s disease (PAI), and chronic mucocutaneous candidiasis (CMC) compared with the general Norwegian APS-1 cohort (gray bars indicate the mean ± SD). (**C**) Illustration of the structure of AIRE. The 4 major subdomains: the homogeneously staining region or caspase recruitment domain/homodimerization domain (CARD/HSR, amino acids 1–105) crucial for the homo- and multimerization of AIRE; the SAND domain (for Sp100, AIRE-1, NucP41/75 or NucP41/75, DEAF-1, amino acids 181–280) needed for protein-protein interaction and DNA-binding; and 2 plant homeodomain (PHD) fingers-type zinc fingers (amino acids 296–343 and 434–475) crucial for proper chromatin binding and DNA interaction. In addition, the AIRE protein contains 4 LXXLL domains that are found on coactivators of nuclear receptors (amino acids 7–11, 63–67, 414–418, and 516–520) and a nuclear localization signal (amino acids 100–189). The gene contains 14 exons represented by rectangles. A schematic representation is given of the splice mutation c.879+1G>A located in the 5′ splice site donor in intron 7. (**D**) Translating RNA from PBMCs to cDNA yielded 3 different outcomes for patients in the AIRE minigene analysis. Only the transcript of AIRE exons 6 and 8 (lane P1_a); a combination of this variant and the full-length AIRE containing both exons 6, 7, and 8 lacking the first 3 bp (lane P1_b); and in some instances only the full-length transcript (lane P1_c) were observed. Data are shown for 1 representative patient and 1 representative control (Cntr). The experiment was performed for all index patients a minimum of 3 times.

Scrutiny of our national registry for organ-specific autoimmune disorders (ROAS) identified 2 female and 2 male patients with APS-1 from 3 different families with late-onset APS-1 (Figure 1A and Table 1). Three of the patients were homozygous for c.879+1G>A (patients 1–3), whereas 1 (patient 4) was compound heterozygous for c.879+1G>A and c.967_979del13, the most common *AIRE* mutation in Norway (Figure 1A). The median age at onset of the first major disease manifestation was 22.5 years (range, 19–43 years) compared with 8.5 (range 0–43 years) in the Norwegian APS-1 cohort. The median number of disease components was 4 (range, 2–6) compared with 5 (range, 1–8) in the Norwegian APS-1 cohort; only 1 patient (patient 3) presented with the 3 major disease components (Figure 1B). All had autoantibodies against IFN-ω and NACHT leucine-rich repeat protein 5 (NALP5), and the median number of autoantibodies was 6 (range, 4–8) of a panel of 14 APS-1–related autoantibodies (17). We studied the children of patients 1 and 4, all being monoallelic carriers of c.879+1G>A or c.967_979del13 and found no APS-1 manifestations or autoantibodies.

### Splicing patterns in APS-1 patients with the c.879+1G>A AIRE mutation.

The splice site mutation c.879+1G>A affects the highly conserved GT dinucleotide motif at the 5′ donor splice site in intron 7 and is thus predicted to affect splicing (Figure 1C). Since *AIRE* transcripts have previously been detected in different immune cell subsets (18–20), we performed RNA extraction and subsequent cDNA synthesis from cultured PBMCs from all patients and matched healthy individuals. The minigene analyses revealed the expected altered splicing pattern in all patient samples but, surprisingly, also the full-length *AIRE* cDNA (Figure 1D), suggesting leakiness of c.879+1G>A as an explanation for the late onset and milder phenotypes.

### AIRE without exon 7 has the potential to induce the expression of AIRE-dependent genes and localizes to the nucleus in typical nuclear speckles.

To investigate the functional capacity of AIRE lacking exon 7 and rule out the occurrence of a dominant-negative effect, we generated a plasmid containing *AIRE* cDNA without exon 7 (*AIRE*^woEX7^). Human thymic 4D6 epithelial cells were transfected with expression vectors containing either WT *AIRE* and/or mutated *AIRE*. c.967-979del13 and c.934G>A (p.C311Y) were used as recessive and dominant-negative controls, respectively. As previously described (21), WT *AIRE* induced high expression of the AIRE-dependent gene *KRT14*, while *AIRE*^woEX7^ induced the expression of *KRT14* at approximately 50% of WT expression levels. We observed little or no transcriptional activity with the *AIRE* mutants c.967-979del13 and c.934G>A. This suggest that the lack of exon 7 led to reduced, although not abolished, transcriptional activity (Supplemental Figure 1A; supplemental material available online with this article; https://doi.org/10.1172/JCI169704DS1). The subcellular location of *AIRE*^woEX7^ was similar to that of WT *AIRE*, revealing typical nuclear speckles (Supplemental Figure 1B). Taken together, these results indicate that *AIRE*^woEX7^retains substantial functional activity in vitro that could explain the milder phenotype.

### The Aire^Ex7–/–^ mutation affects the thymic medullary compartment and developing T cells to a mild degree.

**Figure 2:**
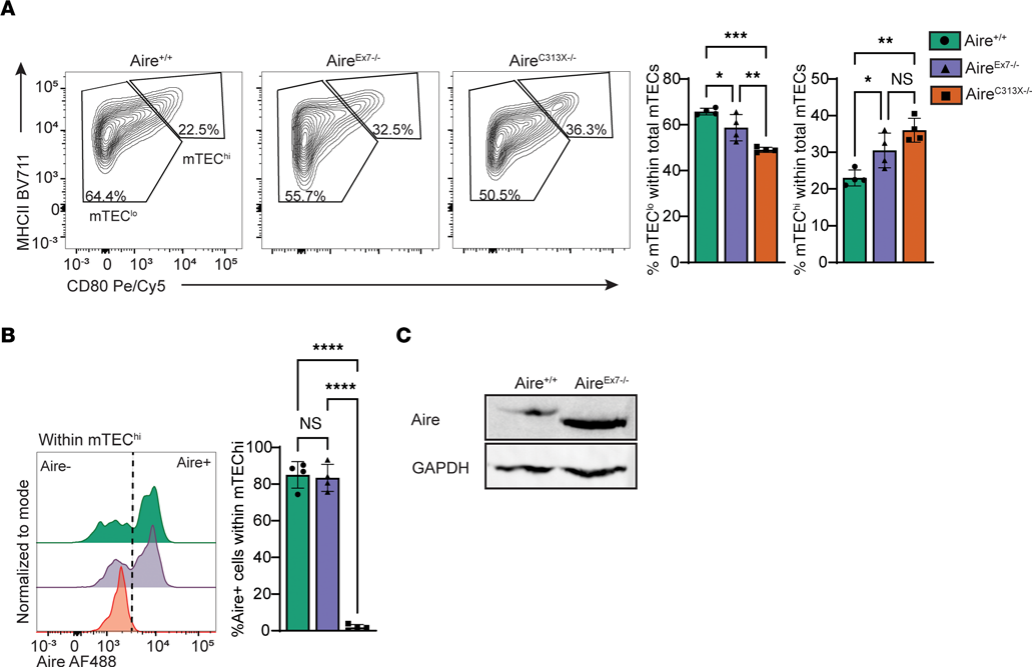
Thymic microenvironment and Aire-regulated gene expression in a mouse model of the AIRE splice mutation. Mice mimicking the human AIRE splice mutation (Aire^Ex7–/–^) and a regular Aire-knockout mouse Aire^C313X–/–^ were analyzed and compared with WT (Aire^+/+^) littermates. (**A**) The frequency of mTEC^hi^ (MHCII^hi^CD80^+^) was increased in mice harboring the 2 Aire mutations compared with WT littermates, with a similar reduction of mTEC^lo^ (MHC II^lo^CD80^–^). (**B**) Aire expression levels in mTEC^hi^ were comparable in both Aire^+/+^ and Aire^Ex7–/–^ mice, whereas Aire^C313X–/–^ mice had no detectable AIRE expression. (**C**) The shorter product of Aire with the Ex7 splice mutation was verified by Western blotting using EpCAM-enriched thymic stroma. Error bars in **A** and **B** represent the SD in 1 of 4 individual experiments (*n* = 3–4 mice in each group). *P ≤ 0.05, **P ≤ 0.01, ***P ≤ 0.001, and ****P ≤ 0.0001, by 1-way ANOVA with multiple comparisons between Aire^C313X–/–^ and Aire^Ex7–/–^ versus Aire^+/+^.

To further characterize the *AIRE* c.879+1G>A mutation under more physiological conditions, we introduced the c.879+1G>A mutation in mice on the B6 background and analyzed the effect on the cellularity of medullary thymic epithelial cells (mTECs) and their capacity to express tissue-restricted antigens (TRAs). When we compared mTEC compartments in the genetically modified mice with those in WT (Aire^+/+^) littermates and with those in previously described B6 Aire^C313X–/–^ mutants (22), we found that both Aire^C313X–/–^ and Aire^Ex7–/–^ mice had significantly reduced frequencies of MHC-II^lo-mid^ CD80^lo-mid^ cells (65.75% ± 1.45%, Aire^+/+^; 58.75% ± 5.75%, Aire^Ex7–/–^; 49.08% ± 1.07%, Aire^C313X–/–^; *P =* 0.0464, Aire^+/+^ vs. Aire^Ex7–/–^; *P =* 0.0002, Aire^+/+^ vs. Aire^C313X–/–^), whereas the frequency of MHC-II^hi^ CD80^hi^ mTECs (mTEC^hi^) was significantly increased (23.00% ± 2.17%, Aire^+/+^; 30.50% ± 4.71%, Aire^Ex7–/–^; 36.03% ± 3.24%, Aire^C313X–/–^; *P =* 0.0361, Aire^+/+^ vs. Aire^Ex7–/–^; *P =* 0.0014, Aire^+/+^ vs. Aire^C313X–/–^) (Figure 2A and Supplemental Figure 2, A–C), although this effect was somewhat smaller than that seen in the Aire^C313X–/–^ mutant. The expression of Aire within mTEC^hi^ was comparable between Aire^+/+^ and Aire^Ex7–/–^ mice (Figure 2B), with slightly quicker migrating bands on the immunoblots, reflecting the reduced molecular weight for Aire without exon 7 (Figure 2C). Aire expression was undetectable in Aire^C313X–/–^ mice, as previously reported (22). Hence, Aire^Ex7–/–^ mice showed adequate expression of Aire, with an increased frequency of mTEC^hi^.

**Figure 3:**
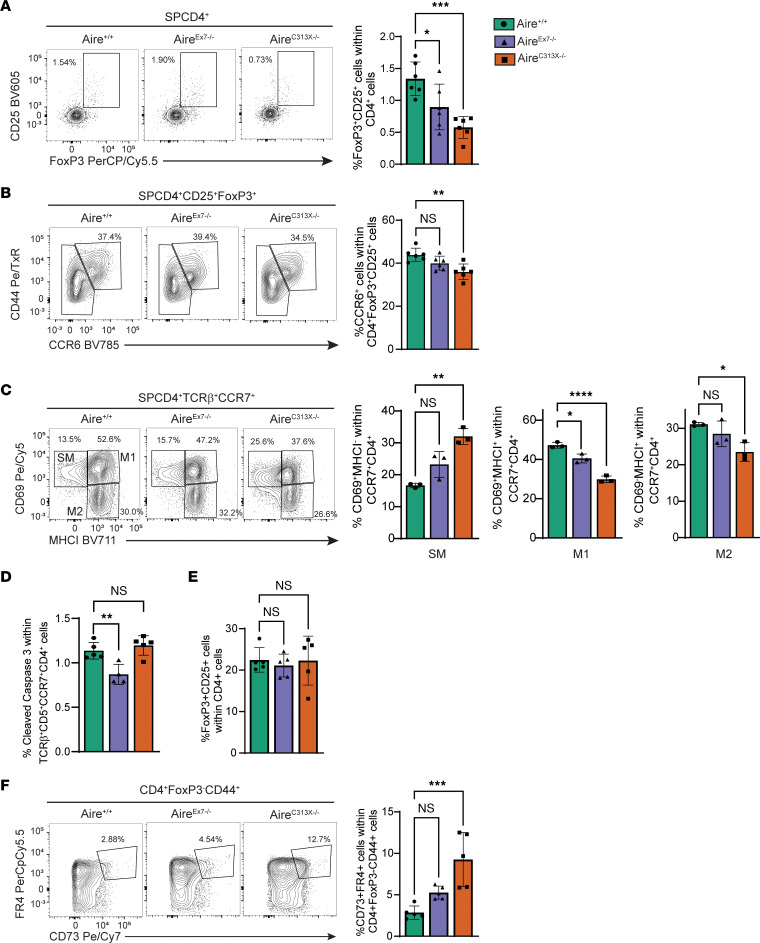
The AIRE splice mutation c.879+1G>A has less of an effect on T cells. (**A**) Thymic Tregs were found to be reduced in Aire^C313X–/–^ and Aire^Ex7–/–^ mice compared with Aire^+/+^ mice. (**B**) Recirculating Tregs were reduced in the thymic compartment only in Aire^C313X–/–^ mice. (**C**) For SPCD4 T cell development, the effect of AIRE loss was greater in Aire^C313X–/–^ mice than in Aire^Ex7–/–^ mice, with a decrease in cells destined for apoptosis, (**D**) as determined by cleaved caspase 3 expression. (**E**) In the spleen, no differences were found within the Treg (CD4^+^FoxP3^+^CD25^+^) compartment. (**F**) Anergic T cells in the spleen, as determined by CD73 and FR3 expression in CD4^+^FoxP3^–^CD44^+^ cells, were increased in the Aire^C313X–/–^ mice. Error bars in **A**–**F** represent SD in 1 of 3 individual experiments (*n* = 3–6 mice in each group). *P ≤ 0.05, **P ≤ 0.01, ***P ≤ 0.001, and ****P ≤ 0.0001, by 1-way ANOVA with multiple comparisons between Aire^C313X–/–^ and Aire^Ex7–/–^ versus Aire^+/+^.

Aire deficiency has previously been linked to defects in the development of thymic Tregs (23–26). Here, both Aire^C313X–/–^ and Aire^Ex7–/–^ mice had a significantly reduced frequency of FoxP3^+^CD25^+^CD4^+^ Tregs compared with Aire^+/+^ mice (1.34% ± 0.26%, Aire^+/+^; 0.89% ± 0.35%, Aire^Ex7–/–^; 0.57% ± 0.17%, Aire^C313X–/–^; *P =* 0.0249, Aire^+/+^ vs. Aire^Ex7–/–^; *P =* 0.0005, Aire^C313X–/–^) (Figure 3A). Furthermore, lack of Aire also affected their homing back to the thymus, as discriminated by the presence of CCR6 (27), a phenomenon we only observed in the Aire^C313X–/–^ mice (43.9% ± 3.05%, Aire^+/+^; 40.05% ± 3.23%, Aire^Ex7–/–^; 36.1% ± 3.59%, Aire^C313X–/–^; *P =* 0.0016, Aire^C313X–/–^) (Figure 3B). Late-stage maturation of single-positive CD4 (SPCD4) T cells can be assessed by CD69 and MHC-I expression (28), and maturation stages 1 and 2 (M1 and M2) were also reduced, mostly in Aire^C313X–/–^ mice, followed by Aire^Ex7–/–^ mice (Figure 3C). Again, we observed an intermediate effect of the Aire^Ex7–/–^ mutation compared with the Aire^C313X–/–^ variant. Interestingly, in the medullary compartment, fewer Aire^Ex7–/–^ CD4^+^ T cells expressed the apoptosis marker caspase 3, suggesting that they received a stronger survival signal than T cells from WT and Aire^C313X–/–^ mice (1.14% ± 0.09%, Aire^+/+^; 0.87% ± 0.11%, Aire^Ex7–/–^; 1.19% ± 0.11%, Aire^C313X–/–^; *P =* 0.0058, Aire^+/+^ vs. Aire^Ex7–/–^) (Figure 3D). Collectively, Aire^Ex7–/–^ mice showed a reduced capacity to develop thymic Tregs but were not as severely affected as the Aire^C313X–/–^ mice.

In the periphery, the levels of mature T cells from the spleen, the frequencies of CD4^+^, CD8^+^, or CD4^+^FoxP3^+^ T cells, or CD4^+^ or CD8^+^ T cells within the memory and naive compartments were equal among the mice (Figure 3E and Supplemental Figure 2, D–L). Anergic T cells lose the ability to produce autocrine growth factors and to proliferate upon antigen recognition and can be assessed using the markers CD73 and FR4. These markers have previously been found to be upregulated in Aire-knockout mice (29). Here, we found an increased frequency of anergic CD4^+^ T cells in Aire^C313X–/–^ mice compared with Aire^+/+^ and Aire^Ex7–/–^ mice (2.85% ± 0.80%, Aire^+/+^; 5.27% ± 0.78%, Aire^Ex7–/–^; 9.25% ± 3.25%, Aire^C313X–/–^; *P =* 0.0005, Aire^C313X–/–^ vs. Aire^+/+^) (Figure 3F), supporting the previously reported data.

### Aire^Ex7–/–^ mice have reduced expression of thymic self-antigens compared with WT littermates.

**Figure 4:**
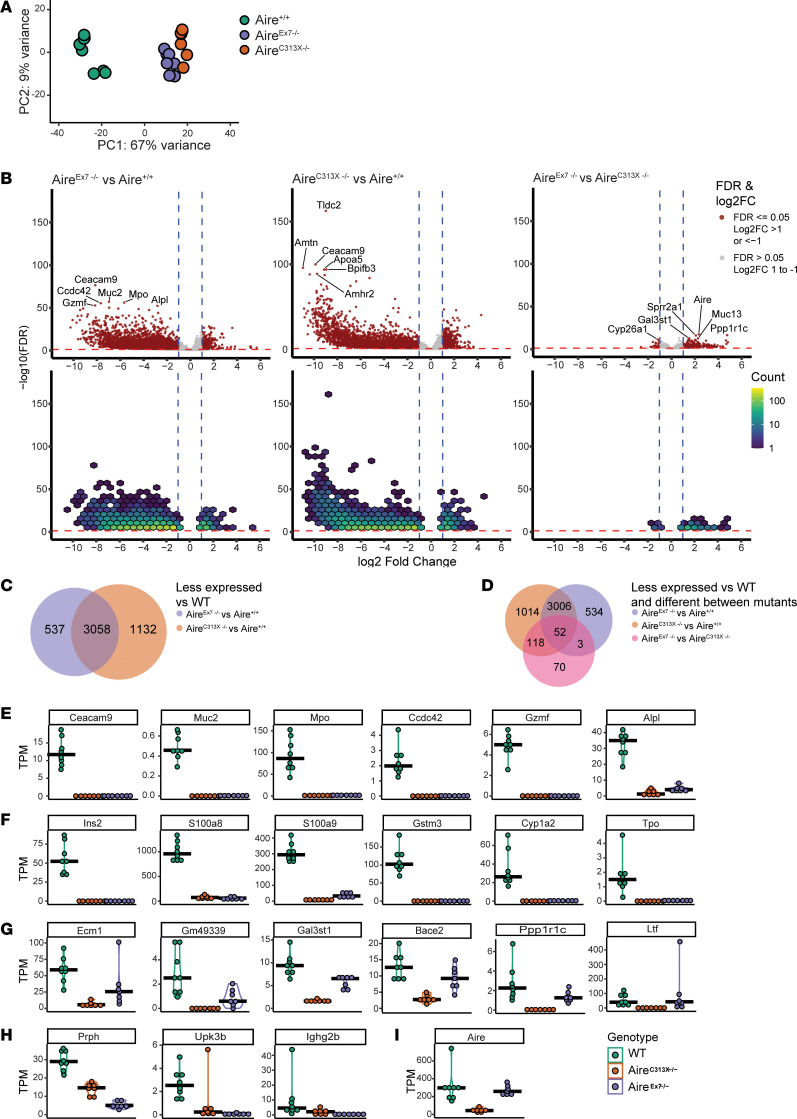
Differential gene expression in mTEC^hi^ populations of Aire mutants versus WT. (**A**) PCA plots showing transcriptomes of mTEC^hi^ from Aire^Ex7–/–^, Aire^C313X–/–^, and Aire^+/+^ mice. (**B**) Volcano and hexbin plots of the differential expression results using DESeq2, looking at each mutant versus Aire^+/+^ or Aire^Ex7–/–^ compared with Aire^C313X–/–^. Significance is shown on the *y* axis as log_10_ (FDR) and relative expression as log_2_ FC on the *x* axis. Hexes are colored according to the number of genes between 1 and 1,000 found within the hex area. Blue lines denote log_2_ FC cutoffs at 1 and –1, and red lines denote the FDR cutoff at 0.05. (**C**) Venn diagram of the overlap of genes expressed at significantly lower levels in Aire^Ex7–/–^ and Aire^C313X–/–^ mice versus Aire^+/+^ mice. (**D**) Venn diagram of genes expressed at significantly lower levels in each mutant versus Aire^+/+^ mice and significantly differentially expressed genes in Aire^Ex7–/–^ versus Aire^C313X–/–^ mice. (**E**) Combination violin and dot plots of absolute expression in transcripts per million (TPM) of the 6 most significantly expressed genes in Aire^Ex7–/–^ versus WT mice. (**F**) TPM of known Aire-regulated genes. (**G**) TPM of the 6 most significantly expressed genes among the 118 genes with significantly lower expression in Aire^C313X–/–^ versus Aire^+/+^ mice and with significantly differential expression in Aire^Ex7–/–^ versus Aire^C313X^^–/–^ mice. (**H**) TPM of the 3 genes with significantly lower expression in Aire^Ex7–/–^ compared with WT mice and with significantly differential expression in Aire^Ex7–/–^ compared with Aire^C313X–/–^ mice. (**I**) Expression of Aire in the 3 groups. (**E**–**I**) Black bars denote the median TPM in mice in each population for each gene.

To investigate the functionality of the Aire^Ex7–/–^ variant and its effect on the expression of TRAs, we performed bulk RNA-Seq of FACS-sorted mTEC^hi^ from Aire^Ex7–/–^, Aire^C313X–/–^, and Aire^+/+^ littermates. Principal component analysis (PCA) plots indicated that the transcriptomes of mTEC^hi^ from both Aire^Ex7–/–^ and Aire^C313X–/–^ mice were distinctly different from those of Aire^+/+^ mice. Principal component 1 (PC1) accounted for 67% of the variance, suggesting that both mutants had a substantial effect on gene expression in mTEC^hi^ (Figure 4A). Differential expression analysis revealed that the ability to induce gene expression was severely impaired in both mutants compared with Aire^+/+^ mice, although fewer genes were significantly different in Aire^Ex7–/–^ mice than in Aire^C313X–/–^ mice compared with Aire^+/+^ mice: a total of 3,595 genes showed significantly lower expression (FDR ≤ 0.05, log_2_ fold change [FC] >1 and <–1) in Aire^Ex7–/–^ mTEC^hi^ and 4,190 genes in Aire^C313X–/–^ mTEC^hi^ compared with Aire^+/+^ mTEC^hi^ (Figure 4B). We detected a higher fraction of genes with lower expression levels in Aire^C313X–/–^ mice versus Aire^+/+^ mice compared with Aire^Ex7–/–^ mice versus Aire^+/+^ mice (Figure 4C), suggesting that Aire^Ex7–/–^ mice retained some residual ability to induce gene expression. Further analysis of the differential expression between Aire^Ex7–/–^ and Aire^C313X–/–^ mice (FDR ≤ 0.05, log_2_ FC >1 and < –1) showed 219 genes with significantly higher expression in Aire^Ex7–/–^ mice, while only 24 genes were expressed at higher levels in Aire^C313X–/–^ mice (Figure 4D). Looking at the overlap of those genes that were expressed at significantly lower levels in only 1 mutant compared with Aire^+/+^ mice, as well as significantly differentially expressed between mutants, we found that 118 genes were expressed at significantly lower levels in Aire^C313X–/–^ mTECs, while 3 genes were expressed at significantly lower levels in Aire^Ex7–/–^ mTECs (Figure 4D). Hence, some TRA expression was retained despite loss of exon 7.

To further illustrate the effect of Aire on gene expression at the individual gene level, we plotted the absolute gene expression of (a) the 6 most significant differentially expressed genes in Aire^Ex7–/–^ compared with Aire^+/+^ mice (Figure 4E), (b) 6 canonical AIRE-regulated genes (Figure 4F), (c) the 6 most significant differentially expressed genes among the 118 genes uniquely less expressed in Aire^C313X–/–^ mice (Figure 4G), and (d) the 3 genes uniquely less expressed by Aire^Ex7–/–^ mice (Figure 4H). Expanded data sets from Figure 4, E and F, as well as known mTEC-related genes and genes expressed at significantly lower levels between Aire^C313X^ and Aire^+/+^ mice as well as between Aire^Ex7–/–^ and Aire^C313X^ mice can be found in Supplemental Figure 3. Overall, more genes failed to be induced in Aire^C313X–/–^ mice than in Aire^Ex7–/–^ mice, while some genes were found to be expressed at low levels in Aire^Ex7–/–^ mice. These findings were also confirmed by quantitative PCR (qPCR) (Supplemental Figure 4A). *Aire* itself was expressed at similar levels in Aire^Ex7–/–^ and Aire^+/+^ mice, whereas Aire^C313X–/–^ mice showed significantly (*P* = 1.17 × 10^–17^) lower expression (Figure 4I). There was no apparent general pattern regarding the absolute expression of the genes significantly downregulated in either of the mutants (Supplemental Figure 4, B–G) or in the types or categories of genes that were able to be induced in these mice. After BioMart conversion of the genes expressed at significantly lower levels into human orthologs and checking them against the Human Protein Atlas data for tissue specificity, we found the distribution to be similar between the uniquely downregulated genes in Aire^C313X–/–^ and Aire^Ex7–/–^ mTECs, with 28% non-tissue-specific genes in Aire^C313X–/–^ mTECs and 27% non-tissue-specific genes in Aire^Ex7–/–^ mTECs, respectively (Supplemental Figure 5A). Combined, these data demonstrate that, even if the Aire^Ex7–/–^ mutant had dramatically impaired transcriptional capacity, it still retained some ability to induce gene expression of TRAs compared with the apparent complete loss-of-function Aire^C313X–/–^ mutant.

### Leaky effects of the exon 7 splice mutation in AIRE.

**Figure 5:**
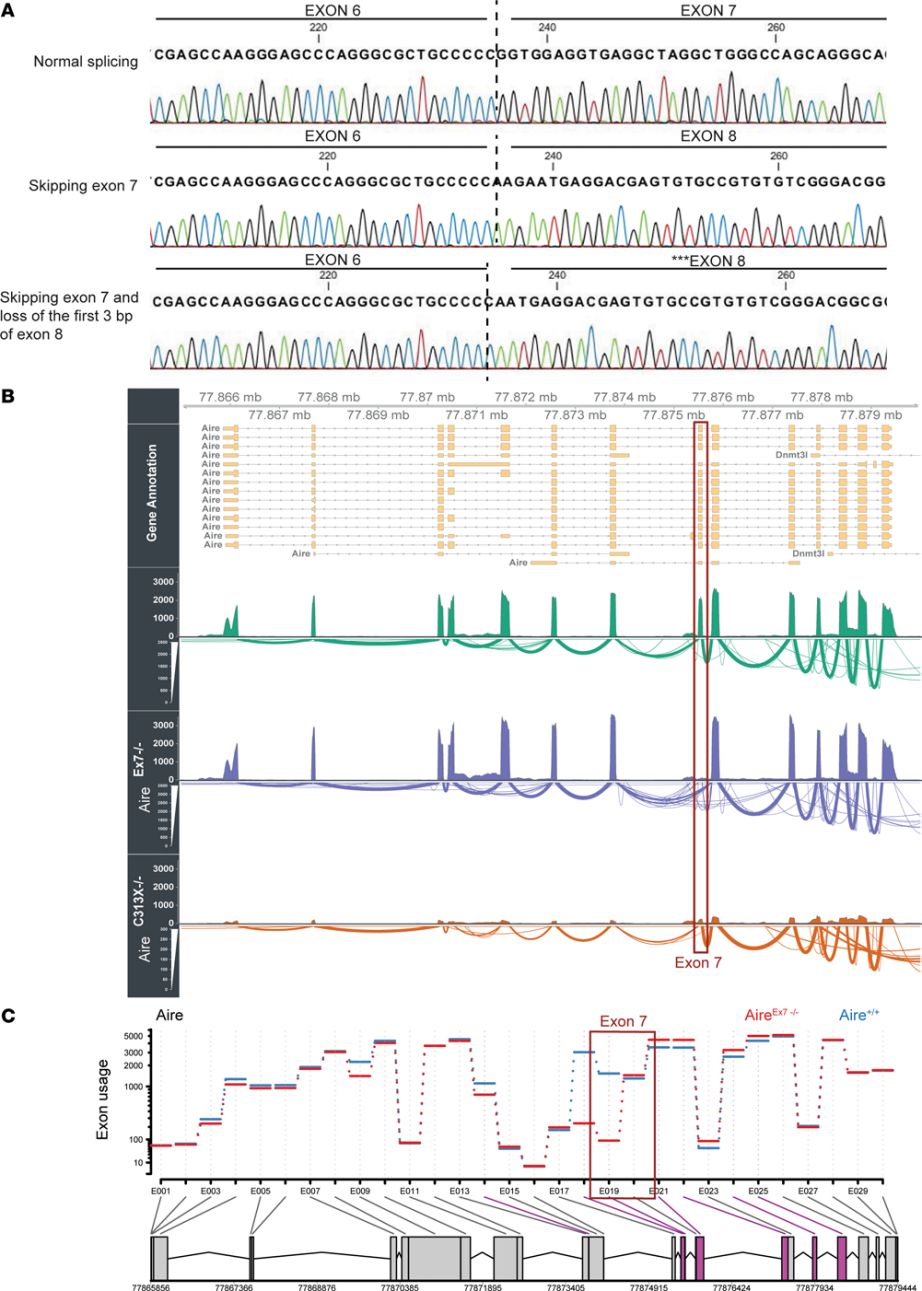
c.879+1G>A leads to incomplete splicing and the production of a small proportion of WT Aire in both mice and humans. (**A**) In PBMCs from patients, 3 different patterns of AIRE expression were observed: normal splicing, skipping of exon 7, and loss of exon 7 and the first 3 bp of exon 8. (**B**) RNA-Seq read coverage and sashimi plot of Aire in 3 representative mice, with Aire^+/+^ mice shown in green, Aire^Ex7–/–^ mice in purple, and Aire^C313X–/–^ mice in orange. (**C**) Differential exon use analysis using DEXseq of Aire in all Aire^Ex7–/–^ mice compared Aire^C313X–/–^ mice. The location of exon 7 is highlighted in dark red.

We next sought to decipher the mechanistic impact of the AIRE exon 7 splice mutation. Most prediction algorithms for splice variants predict a total abolishment of the splice site for this mutation, thus making skipping of exon 7 a highly likely event. The deep neural network–based prediction tool SpliceAI (30) indicates a high probability for loss not only of the donor slice site but also of the acceptor splice site of exon 7, located 81 bp upstream of the mutation. Neither did SpliceAI predict any alternative or cryptic splice sites in the vicinity of the mutation that could lead to frameshifts by retention of the intronic sequence or the introduction of pseudoexons. Skipping of exon 7 is therefore compatible with a retained reading frame of AIRE, leading to a polypeptide without the 27 amino acids comprising exon 7. Surprisingly, we observed both normal splicing and skipping of exon 7, including skipping of exon 7 and the first 3 bp in exon 8, in both cases keeping the reading frame intact. None of these alternative splice patterns were detected in healthy individuals (Figure 5A). The aligned Aire transcripts from RNA isolated from the 3 different mouse strains showed that, although severely reduced, the presence of exon 7 was still detectable in mTECs from the Aire^Ex7–/–^ mice (Figure 5B), and differential exon analysis estimated approximately 10% retained exon usage (Figure 5C). However, it is unclear if any full-length Aire was translated from these transcripts.

### Aire^Ex7–/–^ mice show signs of autoimmunity.

**Figure 6:**
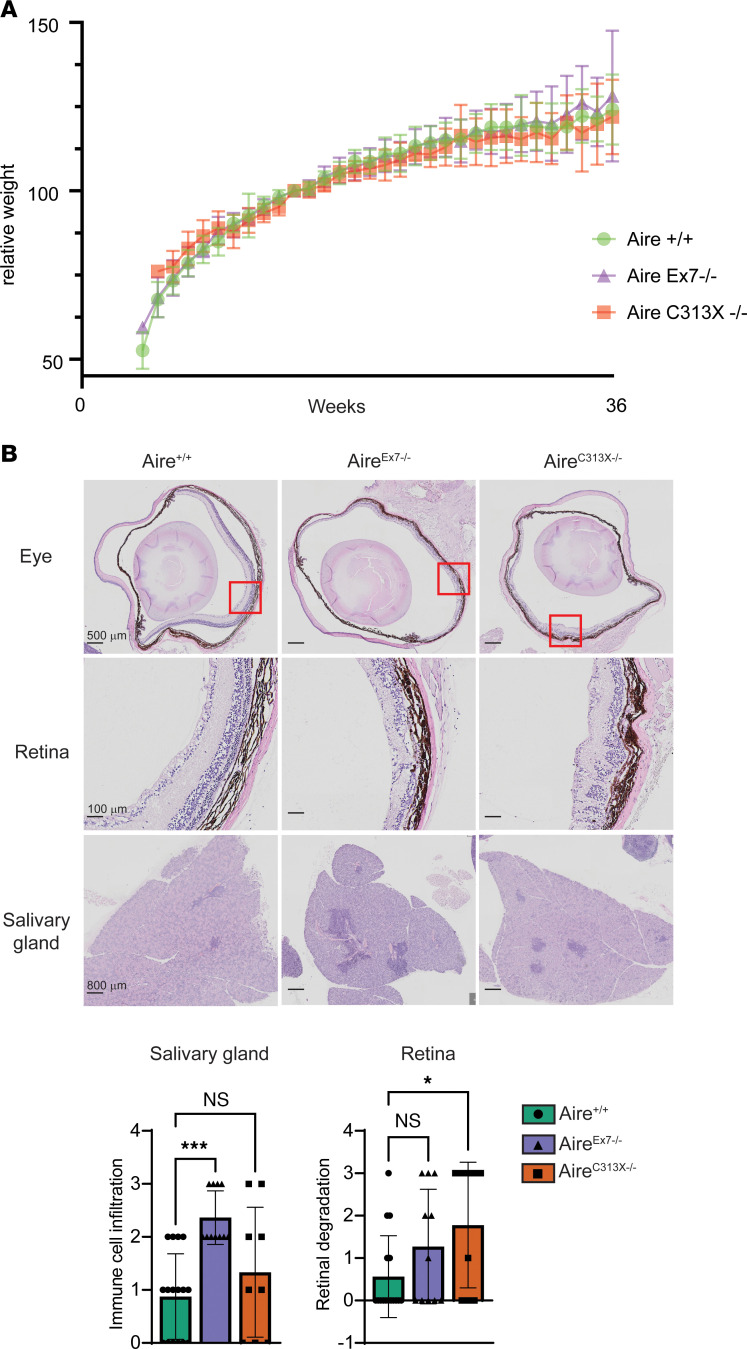
Mice on the c.879+1G>A background develop signs of autoimmunity. (**A**) Weight change curves of Aire^C313X–/–^, Aire^Ex7–/–^, and Aire^+/+^ mice from the earliest possible time point until the end of the experiment. Weight change was calculated for each mouse from its weight at 14 weeks of age. Data are presented as the mean ± SD. One-way ANOVA found no difference in weight between groups at last the recorded weight (*P* = 0.58, *n* = 8–19). (**B**) Infiltration of immune cells was evident in several tissues, most prominently in Aire^C313X–/–^ mice, whereas salivary glands of Aire^Ex7–/–^ mice had severe tissue infiltration. Error bars in **A** and **B** represent the SD (*n* = 9–16 mice per group). *P < 0.05 and ***P < 0.001, by 1-way ANOVA with multiple comparisons between Aire^C313X–/–^ and Aire^Ex7–/–^ mice versus Aire^+/+^ mice.

To test whether the impaired expression of TRA genes in the thymus of the Ex7 mutants translates into an autoimmune phenotype, mice were aged until week 35 and analyzed for signs of autoimmunity. As expected for Aire deficiency on the B6 background, we observed no wasting disease, and both the Aire^Ex7–/–^ and Aire^C313X–/–^mice had weight increases similar to the increases observed in Aire^+/+^ mice (Figure 6A). Since Aire deficiency on the B6 background typically results in autoimmune retinal degradation and high lymphocyte infiltration of the salivary and prostate glands (31), we specifically focused on these 3 tissues. Indeed, we found that both Aire^C313X–/–^ and Aire^Ex7–/–^ mice developed more lymphocyte infiltrations in these organs than did their Aire^+/+^ age-matched controls, albeit the impact of the Ex7 mutation was milder than that of C313X. As expected, other analyzed tissues (lung, prostate, pancreas) were not affected by either of the mutations (Figure 6B and Supplemental Figure 5B). After breeding homozygous Aire^Ex7–/–^ males with heterozygous females, 0.25 litters were recorded, compared with 2.6 litters recorded with heterozygous breeding of the strains, suggesting that the Aire^Ex7–/–^ mice, like the Aire-knockout mice (32), had markedly reduced fertility. Overall, the development of autoimmunity was reflected in the Aire^Ex7–/–^ mice with lymphocyte infiltration in several tissues.

## Discussion

Here, we describe several patients with APS-1 presenting with milder autoimmune disease, who harbor the splice mutation c.879+1G>A in *AIRE* causing loss of exon 7, which was confirmed by observations in a corresponding mouse model. The milder phenotypes can be explained by the presence of full-length AIRE transcripts detected in both mice and patients with APS-1. However, the total AIRE activity was probably severely reduced, as the patients displayed clear APS-1 disease, albeit with later onset and slower disease progression than classical APS-1 (5, 7, 33, 34). Our data illustrate an interesting quantitative association between AIRE activity and the severity of the autoimmune disease, with implications for more common autoimmune diseases like primary adrenal insufficiency, pernicious anemia, type 1 diabetes, and rheumatoid arthritis, for which coding variants in AIRE have been identified in GWAS studies (35–38).

Most human genes contain exons and introns that are spliced together before transcription or cotranscriptionally. The correct identification of exons within the pre-mRNA is essential for splicing and depends on the recognition of a variety of motifs, including the highly conserved GT and AG nucleotides at the 5′ (donor) and 3′ (acceptor) splice sites (39), and the branch site about 15–35 bases upstream of the 3′ splice site. Splicing requires extreme precision because even a single nucleotide addition or deletion will shift the reading frame. The c.879+1G>A mutation is located in the invariant dinucleotide GT at the 5′ splice site donor of intron 7 in *AIRE*, leading to an alternative splice pattern, in which exon 7 is lost but the reading frame is retained. Exon 7 lies between the SAND and PHD1 domains with no described function, but its loss will bring these domains closer together, likely affecting the 3D protein structure (16). Donor splice-site mutations are often located in positions +2 or +1 and are generally more common than mutations located in the splice acceptor site (40). Mutations in the obligate GT and AG dinucleotides virtually always cause exon skipping, cryptic splice-site utilization, or intron retention, resulting in a substantial reduction or absence of normally spliced mRNA (40). Our findings suggest that the c.879+1G>A mutation weakens exon recognition. Thus, both aberrantly and correctly spliced products are made. Surprisingly, we observed 2 aberrantly splice products: the expected skipping of exon 7 and the skipping of exon 7 and loss of the first 3 nucleotides (AAG) of exon 8. A likely explanation for the latter pattern is that the second and third nucleotide (AG) of exon 8 acts as an alternative splice acceptor site, previously described as a potential splicing isoform in mice and in human spleen and bone marrow (41).

Several disease-causing mutations are suggested to affect mRNA splicing (42), which makes splicing mechanisms highly relevant to understanding the molecular pathobiology of genetic diseases, including *AIRE* and APS-1. Functional alternative splicing was previously reported for 2 *AIRE-*mutations: c.463G>A and c.653-1G>A (13, 14). Both mutations cause alternative pre-mRNA splicing by intron retention. Another study involving patients with APS-1 in North America described a patient with the genotype Q173X/IVS9-1G>A. The authors speculate that this splicing mutation, which affects the splice acceptor site of exon 10, could lead to the skipping of exon 10 and thus to the deletion of 60 amino acids from the proline-rich region between the 2 PHD finger motifs, or, alternatively, to the use of a cryptic 3′ splice site in exon 10 (15). However, this was not verified by molecular analyses.

A previous study described the construction of a series of deletion mutants in *AIRE* by systematically removing 1 or more of the functional domains and investigating the stability and subcellular compartmentalization of the corresponding polypeptides (43). One of these was the deletion of exon 7 of *AIRE* studied here. Both the estimated half-life and the intracellular localization of the AIRE polypeptide without exon 7 were comparable to full-length AIRE (43). *AIRE* mRNA without exon 7 also probably escapes degradation by nonsense-mediated mRNA decay, as the reading frame is intact in contrast to Aire^C313X–/–^ (22, 44). In line with this, we observed AIRE transcripts in the patients and Aire expression in Aire^ex7–/–^ mice at both the mRNA and protein levels. Furthermore, we detected the typical nuclear localization and colocalization with WT AIRE in transfected 4D6 cells, in line with previous findings (21).

**Figure 7:**
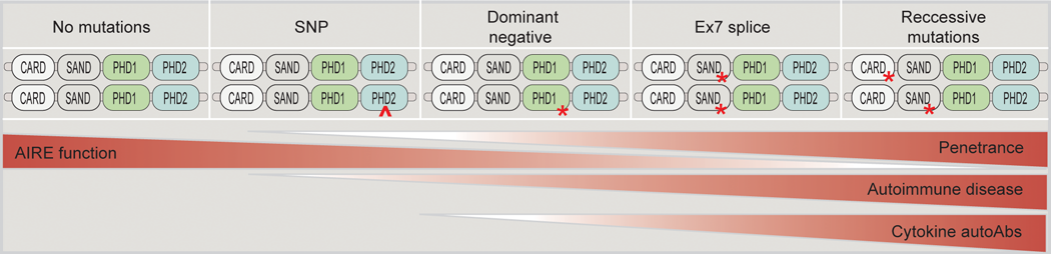
Proposed spectrum of AIRE variants and phenotypical patterns. The range of AIRE, from normal to completely inhibited protein expression with the corresponding type of mutation and phenotypic consequences. A common SNP (p.R471C), dominant-negative mutations, and exon 7 (Ex7) splice mutation give a partially inhibited phenotype, with an increasing level of penetrance.

Here, we demonstrate that Aire^Ex7–/–^mice, as opposed to the Aire^C313X–/–^ mouse model, had the potential to induce transcription of some AIRE-dependent genes. The functional effect of T cell responses to identified Aire-dependent antigens was not studied here but would be an interesting path to explore in the future. As AIRE has been shown to act in a dose-dependent manner (45), this residual AIRE function may contribute to prevent severe disease, thus explaining the delayed disease onset and slower disease progression. Scrutiny of patients with atypical APS-1 has highlighted how genetic variants in *AIRE* confer a broad clinical outcome. The dominant mutations located primarily in the PHD domains of AIRE have been shown to yield mild autoimmune disease with incomplete penetrance within several families (21, 46). In GWAS studies, the AIRE p.Arg471Cys variant has been linked to an increased risk of developing autoimmune Addison’s disease (47), type 1 diabetes (35), and pernicious anemia (36) (Figure 7), while a coding variant in the SAND domain is also associate with Addison’s disease and rheumatoid arthritis (37, 38). As it has been suggested that AIRE-dependent thymic dysfunction may underly the production of autoantibodies against type I IFNs, the spectrum of residual AIRE function might affect the natural protection against infections in these patients (48).

The correlation between protein function and clinical phenotype is demonstrated in several genetic diseases. Duchenne muscular dystrophy and its milder variant Becker muscular dystrophy are both caused by dystrophin gene mutations (49), which often eliminate 1 or more internal exons or alter proper splicing. However, mutations that preserve the reading frame produce a semifunctional protein and cause a mild phenotype, whereas mutations disrupting the reading frame cause a severely truncated protein that would be unstable and result in a severe phenotype (49, 50). Patients with Lesch-Nyhan syndrome (LNS) have disease-causing splicing mutations in the hypoxanthine-guanine phosphoribosyltransferase 1 (*HPRT1*) gene, which normally causes exclusion of exon 4, exon 5, and exon 6, respectively. This genotype results in a complete deficiency of HPRT activity, leading to an error in purine metabolism associated with uric acid overproduction and neurological manifestations (51). Similar to what we found, patients with mild LNS lacking some of the typical symptoms are reported to have a minor amount of normally spliced *HPRT* mRNA despite disease-causing mutations (51). Mutations that alter the ratio of alternatively spliced isoforms can also influence the disease phenotype. A well-characterized example is frontotemporal dementia and Parkinsonism linked to chromosome 17 (FDTP-17), which is caused by mutations in the microtubule-associated protein tau (*MAPT*) gene encoding the tau protein (52). These mutations alter the ratio of the 2 tau isomers by disrupting the splicing of exon 10, which is the causative event in tau aggregation and onset of disease (52). Given the above findings, associations between genotype, altered mRNA splicing, protein function, and phenotypic expression seem to be well established for several genetic disorders. Our data indicate that AIRE^Ex7–/–^ transcripts are translated into a hypomorphic AIRE protein with the capability to induce AIRE-regulated genes, resulting in a milder phenotype in patients.

In conclusion, we report an AIRE splice mutation (c.879+1G>A) found in 4 Norwegian patients with APS-1 who have a milder phenotype compared with classical APS-1. The pathogenic role in altering *AIRE* pre-mRNA splicing was confirmed by minigene analyses and subsequent cDNA sequencing. The altered *AIRE* transcripts (skipping exon 7) produced a protein with residual AIRE function with a normal nuclear localization. Importantly, the occurrence of full-length transcripts and a residual function of the AIRE protein lacking exon 7 explain the milder phenotype observed in these patients. Taken together, studies of the functional mutations at the genomic DNA level will widen the molecular spectrum of APS-1 and provide valuable insights into the molecular mechanisms underlying its pathophysiology.

## Methods

### Study design.

Sample sizes for the patients were determined by their AIRE mutation, hence, all patients in our registry with this mutation were included. Healthy controls were always analyzed alongside patient material. For the in vitro experiments, we chose to use 3 independent experiments, each with 3 parallels. For the mouse work, we used between 3 and 6 mice in each group in total, a minimum of 2 independent experiments were executed for all experiments, and mice from all 3 groups were always included in each individual experiment. The scoring of the tissue infiltrates was done in a blinded fashion by 4 individual scientists, whereby the scoring was determined before the genetic background was revealed. The objective of the study was to determine the functional outcome of the exon 7 splice mutation identified in the patients and to further elaborate AIRE’s function in negative selection.

### Patients and clinical data.

The patients with APS-1 presented were previously described as part of the Norwegian cohort and included in our National Registry of Autoimmune Diseases (7, 53, 54). The cohort includes 4 patients from 3 families, with the first major APS-1 manifestations diagnosed between 19 and 43 years of age. An overview of their characteristics is provided in Table 1. The patients were followed at least annually by an endocrinologist. Healthy age- and sex-matched controls were recruited from the local blood bank at Haukeland University Hospital.

### Isolation of PBMCs, RNA isolation, and cDNA synthesis.

PBMCs were isolated from heparinized blood by Ficoll-Paque PLUS (GE Healthcare) density gradient centrifugation and cultured in RPMI-1640 medium with 10% human AB serum (MilliporeSigma) and 1% penicillin-streptomycin (MilliporeSigma) for 72 hours. Total RNA was then isolated (RNeasy Mini Kit, QIAGEN) and stored at –80°C. Reverse transcription reaction was performed using High-Capacity cDNA Reverse Transcription Kit (Applied Biosystems), strictly according to the manufacturer’s instruction.

### PCR and cDNA sequencing of AIRE in PBMCs.

A minigene construct, spanning the splice site affected by the mutation c.879+1G>A, and with a total length of 5 exons (exon 5 to exon 10) was designed to map mRNA transcripts of *AIRE* in PBMCs from patients and healthy individuals. The constructs with their flanking exon-intron boundaries of the *AIRE* gene were amplified by PCR using AmpliTaq 360 DNA Polymerase (Applied Biosystems) and the following program on a thermal cycler (GeneAmp PCR System 9700, Applied Biosystems): 98°C for 10 minutes, followed by 10 cycles of 98°C for 15 seconds, 69°C for 15 seconds, and 72°C for 1 minute, and 42 cycles of 95°C for 15 seconds, 50°C for 15 seconds, and 72°C 1 minute, followed by 78°C for 1 minute and, finally, 4°C. The PCR product was purified with ExoSap (GE Healthcare) and sequenced using the BigDye Terminator version 1.1 Cycle Sequencing Kit (Applied Biosystems). The primer sequences (5′–3′) were as follows: forward primer, GATTCAGACCATGTCAGCTTC and reverse, GCAGCACGTCCGTACCATCTC (MilliporeSigma), both of which were used for amplification and the sequencing reaction. PCR products were visualized on a simulated gel system following the instructions of the manufacturer (Agilent Technologies).

### Plasmid construction.

WT *AIRE* and *AIRE* without exon 7 (AIRE^woEX7^) in pCMV6 vectors with C-terminal Myc- DDK Tag were purchased from OriGene and Thermo Fisher Scientific, respectively, and the mutations c.769C>T, p.(Arg257Ter) and c.932G>A, p.(Cys311Tyr) were created using the QuikChange II Site-Directed Mutagenesis Kit (Agilent Technologies) to mimic known recessive and dominant APS-1–causing mutations. The plasmid pEGFP-C1 containing WT *AIRE* was a gift from Ismo Ulmanen (Department of Molecular Medicine, National Public Health Institute, Finland). All plasmids were amplified using TOP10-competent *E. coli* (Thermo Fisher Scientific) and purified with the QIAprep Spin Miniprep Kit (QIAGEN), and their correct sequences were confirmed by sequencing (3730 DNA Analyzer, Applied Biosystems).

### Cell culturing, transfection, and RNA extraction.

The transfection of cells and RNA extraction were mainly executed as previously described (21). In brief, human thymic 4D6 epithelial cells, a gift from Christophe Benoist (Harvard Medical School, Boston, Massachusetts, USA) (55, 56) were cultured in RPMI 1640 (Lonza) supplemented with 10% FBS (Thermo Fisher Scientific), 10 mM HEPES buffer (Lonza, Basel, Switzerland), 1% nonessential amino acids (Lonza), 2 mM l-glutamine (Lonza), 100 U/mL penicillin (MilliporeSigma), and 100 μg/mL streptomycin (MilliporeSigma) at 37°C with 5% CO_2_. Cells were then plated in a 6-well plate at a density of 5 × 10^5^ cells per well and incubated overnight. Samples (2.5 μg) of the pCMV6 plasmids were mixed with 10 μL FuGene HD transfection reagent (Promega) to a total volume of 160 μL supplemented with RPMI 1640 (without penicillin or streptomycin) and incubated for 5 minutes at room temperature before being added to the cells. Cells were incubated for another 24 hours before total RNA was extracted (RNeasy Mini Kit, QIAGEN). cDNA was prepared from 1 μg total RNA (High-Capacity RNA-to-cDNA Kit, Applied Biosystems).

### Assay of AIRE-regulated genes.

The ability of transfected AIRE plasmids to induce the expression of selected AIRE-regulated genes and control genes was measured as previously described (21). In summary, the AIRE-regulated genes were analyzed by qPCR using the following primers and probes (Applied Biosystems): keratin 14 (*KRT14*) (Hs00265033-m1) and IGF-like family member 1 (*IGFL1*) (Hs01651089-g1), while the expression of *AIRE* itself was tested with *AIRE* (Hs01102906-g1 and Hs01102908-g1). The results were compared with β2-microglobulin (*B2M*) (4333766) as an endogenous control and with the AIRE-independent genes cyclin H (*CCNH*) (Hs00236923_m1) and protein arginine methyltransferase 3 (*PRMT3*) (Hs00411605_m1). Data sets were normalized to *B2M.* Ct values from cells transfected with pCMV6 plasmids harboring the current mutation or the empty pCMV6 plasmid were then subtracted from the Ct values obtained after transfection with pCMV6 harboring WT *AIRE.* The fold difference was calculated as 2^ [Ct (target gene) – Ct(B2M)] – [Ct (test sample) – Ct (calibrator sample)], with the test sample defined as the current mutation and the calibrator as WT *AIRE.* The assays were repeated 2–4 times ,and the results are shown as the mean values of these replicates.

### Immunofluorescence.

4D6 cells were plated in a 6-well plate containing 18 mm sterile cover slips at a density of 5 × 10^5^ cells per well and incubated overnight, transfected with the pCMV6 plasmids and/or the human EGFP-*AIRE* fusion plasmid as described above, and incubated for another 24 hours. Coverslips with cells were then washed with PBS (MilliporeSigma) and fixed with 4% formaldehyde (MilliporeSigma) in PBS for 7 minutes at room temperature and again washed with PBS. Cells were permeabilized with 0.5% Triton-X (MilliporeSigma) in PBS for 15 minutes at room temperature, washed in PBS, and incubated for 1 hour at room temperature with 10% FBS (Thermo Fisher Scientific) in PBS. Another washing step was done before staining with phycoerythrin-conjugated anti-DYDDDDK (clone L5, BioLegend) in 1% BSA (MilliporeSigma) in PBS for 1 hour at room temperature. Then, the coverslips were washed in PBS and water and attached to SuperFrost microscope slides (Thermo Fisher Scientific) using ProLong Gold antifade reagent with DAPI (Invitrogen, Thermo Fisher Scientific). Slides were incubated for 24 hours in the dark at room temperature and analyzed using a Leica SP5 microscope.

### Mouse models.

B6.Aire^C313X^ (22) and B6.Aire^Ex7^ mice were generated in the transgene mouse facility at The Weizmann Institute of Science, using CRISPR/Cas9 genome editing in isolated 1-cell embryos from C57Bl/6, mice (Supplemental Figure 6 and Supplemental Table 1). Genotyping of mice with splice or point mutations was conducted using custom TaqMan (Thermo Fisher Scientific) SNP Genotyping assays (Supplemental Table 1). Mice were transferred to the animal facility at the University of Bergen and were provided with standard rodent chow and autoclaved water ad libitum. Four- to 7-week-old mice were used in all experiments unless stated otherwise.

### Immunohistochemistry.

Mouse tissues were fixated in 10% formalin at room temperature (RT) for 24 hours before being further processed. Paraffin-embedded tissues were sectioned, stained with H&E, and imaged with an Olympus VS120 slide scanner. Images were assessed with QuPath software (57) and scored according to the following categories: 0 = no infiltration/retinal degradation; 1 = little infiltration/retinal degradation; 2 = moderate infiltration/retinal degradation; and 3 = severe infiltration/retinal degradation.

### SDS-PAGE and immunoblot analysis of Aire.

EpCAM-enriched thymic stromal cells from 2–3 thymi from each genotype were lysed in RIPA buffer (150 mM NaCl, 50 mM Tris-Cl, pH 8.0, 5 mM EDTA, 1% IGEPAL CA-360, 0.5% sodium deoxycholate, and 0.1% SDS) complemented with complete proteinase inhibitors (539134, Calbiochem). Proteins were resolved on 10% SDS-PAGE and electrotransferred onto nitrocellulose membranes. Membranes of samples derived from thymus were treated with Pierce Western Blot Signal Enhancer (21050, Thermo Fisher Scientific) before nonspecific binding blockade performed in a solution of 5% milk in 1× PBS supplemented with 0.05% Tween-20. All incubations with antibodies/serum were done overnight. Signals were detected with an EZ-ECL Kit (Biological Industries) or Immobilion Crescendo Western HRP Substrate (WBLUR0500; MilliporeSigma) and were captured using the ImageQuant TM-RT ECL image analysis system (GE Healthcare). Quantification of WBs was done using Image Lab Software 6.1 (Bio-Rad). The following antibodies were used: polyclonal anti–rat AIRE SAND antibody (a gift from Diane Mathis, Harvard Medical School, Boston, Massachusetts, USA), anti-GAPDH antibody (MAB374; MilliporeSigma), HRP-conjugated anti-rat monoclonal IgG light chain antibody (112–035-175; Jackson ImmunoResearch), and HRP-conjugated goat anti–mouse polyclonal antibody (115–035-003; Jackson ImmunoResearch).

### Isolation, flow cytometry, and sorting of mTEC^hi^.

Thymic epithelial cells were isolated from 4-week-old mice by enzymatic digestion of thymic lobes using 0.30 mg/mL Liberase TM Research grade (Roche) and 75 μg/mL DNase I (PanReac AppliChem). Cells were counted and stained with anti-CD45 microbeads (Miltenyi Biotec) for 15 minutes at 4°C before depletion using an LD column (Miltenyi Biotec) to remove the thymocytes. The cells were subsequently stained with the surface markers for 20 minutes at 4°C. The following panel was used: CD86 phycoerythrin/Cyanine 7 (Pe/Cy7), CD80 Pe/Cy5, Ly51 Pe, CD45 AF700, MHC-II BV711, EpCam BV605, UEA biotin, and streptavidin FITC or BV785 (when staining for AIRE) (see Supplemental Table 1 for antibody specifics and Supplemental Figure 7A for the gating strategy). To assure proper segregation of mTEC^hi^ and mTEC^lo^, the panel was established including an antibody recognizing AIRE (AF-488) using the Foxp3 Transcription Factor Staining Buffer Set (Thermo Fisher Scientific) according to the manufacturer’s instructions. Intracellular stainings were performed for 60 minutes at 4°C in the dark. DAPI (BioLegend) was added as a dead cell marker. Cortical TECs (cTECs), mTEC^hi^, and mTEC^lo^ were sorted on a BD FACS Symphony S6 instrument, directly into 150 μL RNeasy micro lysis buffer (QIAGEN), and RNA was extracted immediately with the RNeasy Plus Micro Kit according to the manufacturers standard protocol (QIAGEN).

### RNA-Seq of mTEC^hi^.

cDNA was made from RNA and amplified using the SMART-Seq version 4 Ultra Low Input RNA Kit according to the manufacturer’s protocol (Takara Bio). Undiluted RNA samples from 2,000–8,000 cells were used for cDNA amplification with 14 cycles (Supplemental Table 2). The cDNA was purified by the Agencourt AMPure XP Kit (Beckman Coulter) and the quality assessed by TapeStation (High Sensitivity D5000 ScreenTape kit) according to the manufacturer’s protocols (Agilent Technologies). The samples were stored at –20°C before use.

Library preparation was performed using the Nextera XT DNA Library Preparation kit and the Nextera XT Index kit according to the manufacturer’s protocol (Illumina). The input cDNA was diluted according to the results from the TapeStation (Supplemental Table 2), and unique combinations of i5 (5 μL) and i7 (5 μL) index adapters were added to each respective sample. The libraries were assessed by TapeStation High sensitivity D5000 ScreenTape (Agilent Technologies), consisting of fragments mostly of 250–1,500 bp, with a peak of 600–1,000. Sequencing of the products was performed at the Bergen Genomics core facility, using a HiSeq 4000 sequencer according to the Illumina TruSeq Stranded mRNA protocol.

### RNA-Seq alignment.

Quality control of fastq files was performed using FastQC (version 0.11.9, https://www.bioinformatics.babraham.ac.uk/projects/fastqc/) and MultiQC (version 1.12) (58). Fastq files were subsequently aligned using the Kallisto pseudoaligner (version 0.46.2) (59) to the GRCm39 release 103 reference genome provided by Ensembl (60). All subsequent analyses were performed using R (version 4.0.2, R Core Team, 2020) and Rstudio (version 1.4.1103, RStudio Team, 2021), in which tximport (version 1.18) (61) was used to import Kallisto output files into R and aggregate transcript data to the gene level using the “mmusculus_gene_ensembl” gene annotation data accessed from biomaRt (version 2.46.3) (62)).

### Differential expression analysis.

Differential expression analysis was performed with DESeq2 (version 1.30.1) (63) using the design formula “~batch + genotype,” with each Aire-mutant mouse population compared separately with WT mice and Aire^Ex7–/–^ mice compared with Aire^C313X–/–^ mice. Significance testing was performed using a likelihood ratio test with the reduced formula “~batch” and a FDR threshold of 5%. A log_2_ FC shrinkage analysis was performed using the ASHR package (version 2.2-47) (64). For robust removal of genes found differentially expressed due to single-sample outliers from the analysis, the max Cook’s distance (63) was set to discard genes with a distance of 3 or greater using the Cooks Cutoff variable. Genes with a log_2_ FC value between 1 and –1 were also removed. Results tables were generated using tidy data principles with the tidyverse package (version 1.3.1, 10.21105/joss.01686), and volcano, hexbin, violin, and dot plots were generated using ggplot2 (version 3.3.5, ISBN 978-3-319-24277-4). The color scales used were generated using the packages RColorBrewer (version 1.1-2, http://colorbrewer2.org/) and Viridis (version 0.6.2, 10.5281/zenodo.4679424). Plots were also generated with the help of the additional packages ggrepel (version 0.9.1), cowplot (version 1.1.1), ggtext (version 0.1.1), and scales (version 1.1.1). Venn diagrams were generated using the VennDiagram (version 1.7.0) package but plotted in ggplot using draw_grob from cowplot. Humanizing the mouse genes was performed with the “mmusculus_gene_ensembl” gene annotation data accessed from biomaRt (version 2.46.3) (62), and the humanized mouse genes were compared with the Human Protein Atlas data on “Tissue Specific,” “Group Specific,” and “Tissue Enhanced” genes (65).

### Differential exon use analysis and read distribution visualization.

Fastq files were aligned to the GRCm39 release 103 reference genome using the STAR aligner (version 2.7.7a) (66) in order to generate BAM files for analysis. STAR was run according to the standard options used by the ENCODE project. In R, the package ensembldb (version 2.14.1) (67) was used with the ensDbFromGtf function to generate a EnsDB annotation object from a GRCm39 release 103 reference GTF file. The genomic coordinates for exons were extracted using the exonicParts function of the GenomicFeatures package (version 1.42.3) (68). In order to quantify exon use in the BAM files, the summarizeOverlaps function from the GenomicAlignments package (version 1.26.0) (68) was used with the exon coordinates previously generated to quantify reads within exons. SummarizeOverlaps was run with the options “mode=”Union”, singleEnd=FALSE, ignore.strand=F, inter.feature=FALSE, fragments=TRUE”. To easily work with the SummarizedExperiment object, the tidySummarizedExperiment package (version 1.0.0) was used. Differential exon use analysis was performed on the comparison of the Aire^Ex7–/–^ samples versus WT by using the DEXseq package (version 1.36.0) (69) with the design formula “~ batch + sample + exon + genotype:exon”. Coverage and sashimi plots for read distribution within the *Aire* gene were generated using the Gviz package (version 1.34.1) (70) with the previously generated BAM files and the EnsDB annotation reference.

### T cell analysis by flow cytometry.

T cells from mouse thymus or spleen were analyzed using flow cytometry. Briefly, the organs were harvested, and the immune cells were released from the tissue by gently straining the tissue through a 70 μm filter using the plunger from a 5 mL syringe. For the spleen samples, cells were treated with 3 mL RBC lysis buffer (Thermo Fisher Scientific) for 5 minutes and 2 μL Purified Rat Anti-Mouse CD16/CD32 (Fc Block) (BD Biosciences) was added before counting and subsequent staining. Staining for cell-surface antigens was performed at 4°C for 20 minutes in the dark. The thymocytes analyzed lacked the expression of CD11b, CD11c, Gr1, CD19, CD49b, F4/80, NK1.1, GL3, and Ter119 (lineage negative). The following panels were used: SM, M1 and M2, antibodies recognizing CCR7 BV421, CD4 APC/Cy7, CD8 FITC, CD69 Pe-Cy5, TCRβ Pe, MHC-1 BV711, cleaved caspase 3 AF647 (Supplemental Figure 7B). The following Treg panel (thymic and peripheral) was used: TCRβ Pe, CD4 APC-Cy7, CD8a FITC, CD25 BV605, FoxP3 PerCP/Cy5.5, and CCR6 BV785 (Supplemental Figure 7, C and D). The following memory/naive panel was used: CD4 APC-Cy7, CD8a FITC, B220 BV650, CD44 phycoerythrin/Texas Red (Pe/TxR), CD62L PE-Cy7, TCRβ Pe, CD25 BV605, FR4 PerCP/Cy5.5, and CD73 PE/Cy7 (Supplemental Figure 7E) (see Supplemental Table 1 for details on the antibodies used). Where needed, staining for CCR6 and CCR7 was performed for 30 minutes at 37°C in a water bath, directly followed by the addition of other cell-surface stains. For the identification of intracellular markers (Foxp3, Helios), the Foxp3 Transcription Factor Staining Buffer Set (Thermo Fisher Scientific) was used according to the manufacturer’s instructions. Intracellular stainings were performed for 60 minutes at 4°C in the dark. Cell viability was measured using the LIVE/DEAD Fixable Aqua Dead Cell Stain Kit (Thermo Fisher Scientific) according to the manufacturer’s instructions. The panels were acquired on a BD LSR Fortessa at the Flow Cytometry Core Facility of the University of Bergen and analyzed using FlowJo version 10 (BD Life Sciences).

### Statistics.

Two-tailed Student’s *t* tests were performed with GraphPad Prism version 9 (GraphPad Software), and the level of significance was defined as a *P* value of less than 0.05. When comparing 3 groups, 1-way ANOVA was used with Dunnett’s multiple-comparison test.

### Study approval.

The Regional Committee for Medical and Health Research Ethics approved the study (approval no. REK 2009/2055 and 2013/1504), and all participants provided written informed consent. All animal handling and experiments were approved by the Norwegian Animal Research Authority and conducted in strict accordance with the European Convention for the Protection of Vertebrates used for Scientific Purposes (FOTS no. 13570).

### Data availability.

RNA-Seq data were deposited in the European Nucleotide Archive (ENA) (https://www.ebi.ac.uk/ena; accession number PRJEB57099). The Supplemental Supporting Data Values file contains all values for data points shown in graphs and values behind the reported means. Further information and requests for resources and reagents should be directed to and will be fulfilled by the corresponding author.

## Author contributions

BEO and ESH organized the study. ØB and ESH recruited the patients and described their clinical history. ØB, ASBW, AH, PMK, SJ, EB, and BEO characterized the patients genetically and biochemically. SBD, RHK, JA, and YGAS generated mouse models. YGAS, BEO, AHB, ASBW, LB, and EB characterized and maintained the mouse models. BEO and ESH led the writing of the manuscript, and all authors provided critical feedback and helped shape the research, analysis, and conclusions of the manuscript.

## Supplementary Material

Supplemental data

Supporting data values

## Figures and Tables

**Table 1:**
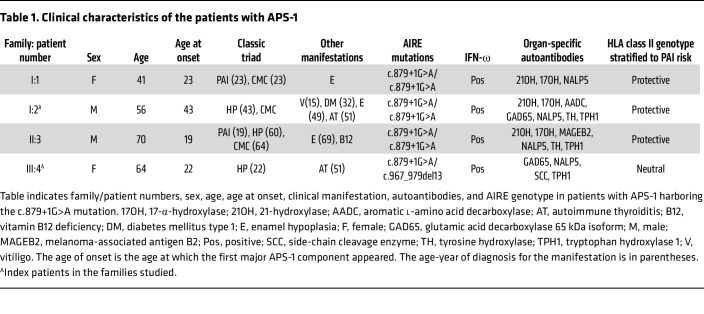
Clinical characteristics of the patients with APS-1

## Version 1 11/01/2023

Electronic publication
