# Supplementary material for: A partial form of AIRE deficiency underlies a mild form of autoimmune polyendocrine syndrome type 1
Source: J Clin Invest. 2023 Nov 1;133(21):e169704. doi: 10.1172/JCI169704 (PMC10617782; doi:10.1172/JCI169704)
Supplement: Supplemental data [file jci-133-169704-s095.pdf]

Supplemental Table 1

**Kit and reagents**		**Manufacturer**	**Cat. Number**
Liberase TM Research Grade		Roche	5401119001
Dnase I		PanReac AppliChem	A3778,0010
CD45 MicroBeads, mouse		Miltenyi Biotec	130-052-301
Rneasy Micro Kit		Qiagen	74004
High-Capacity RNA-to-cDNA™ Kit		Thermo Fisher Scientific	4387406
**Antibodies**	**clone**	**maufacturer**	**Cat. Number**
Pe/Cy7 anti-mouse CD86	GL-1	BioLegend	105014
Pe/Cy5 anti-mouse CD80	16-10A1	BioLegend	104712
Pe anti-mouse Ly-51	6C3	BioLegend	108308
AF700 anti mouse CD45	30-F11	BioLegend	103128
BV711 anti-mouse I-A/I-E	M5/114,15.2	BioLegend	107643
BV605 anti-mouse CD326 (EpCam)	G8.8	BioLegend	118227
UEA I, biotinylated		Vector Laboratories	B1065
AF-488 Anti-Mo Aire	5H12	eBioscience	53-5934-82
BV785 anti-mouse CD196 /CCR6)	29-2L17	BioLegend	129823
BV421 anti-mouse CD197 (CCR7)	4B12	BioLegend	120120
BV650 anti-mouse CD45R/B220	RA3-6B2	BioLegend	103245
BV605 anti-mouse CD25	PC61	BioLegend	102035
BV421 anti-mouse CD62L	MEL-14	BioLegend	104435
Pe/TR anti-mouse CD44	IM7	BioLegend	103023
PerCp/Cy5.5 anti-mouse FoxP3	4B10	BioLegend	644805
APC/Cy7 anti-mouse CD4	GK1.5	BioLegend	100413
FITC anti-mouse CD8a	53-6.7	BioLegend	100706
PerCP7Cy5.5 anti-mouse FR4	12A5	BioLegend	125007
Pe/Cy7 anti-mouse CD73	TY/11.8	BioLegend	127223
BV711 anit-mouse MHCI	M5/114.15.2	BioLegend	107643
Pe anti-mouse TCRb	H57-597	BioLegend	109207
Pe-Cy5 anti-mouse CD69	H1.2F3	BioLegend	104509
AF647 Cleaved Caspase 3	D3E9	cell signaling	96025

CRISPR (Aire Ex7)			
sgRNA	GGACTTACCTGGTTAACCTG		
ssODN	gcagaaagtgggccagcagtgtggggttcctccccttccatccctccccagtgagccccaagtcaaccag ATAAGTCCCAAGAAGGGGGTGGGGGTGGGGGAACCAGGATATGGAGGGCAGCTCCCTTCC T		
**Primers**	**5'-3' sequence**		
Human AIRE exon 5_Fw	gattcagaccatgtcagcttc		
Human AIRE exon 10 Rv	gcagcacgtccgtaccatctc		
**Genotyping assays**	**Name**	**Cat. #**	**5'-3' sequence**
C313x	Aire_C313X_F	ANU6663	GTCAGAAGAACGAGGATGAGTGT
	Aire_C313X_R	ANU6663	CAGGCAAGCCAGGTGGAA
	Aire_C313X_Vic	ANU6663	ACAGCCGTCACAACA
	Aire_C313X_Fam	ANU6663	ACAGCCGTCTCAACA
Splice Ex7	Aire-ex7_F	ANDJ2XD	TTCCATCCCTCCCCAGTGA
	Aire-ex7_R	ANDJ2XD	CTGCCCTCCATATCCTGGTT
	Aire-ex7_Vic	ANDJ2XD	AACCAGGTAAGTCCC
	Aire-ex7_Fam	ANDJ2XD	AACCAGATAAGTCCC
**TaqMan probes**	Krt1	Mm00492992_g1	
	Ecm1	Mm00514634_m1	
	Upk3b	Mm00558406_m1	
	Aire	Mm00477461_m1	
	Ins2	Mm00731595_gH	
	S100a9	Mm00656925_m1	

Supplemental Table 2

Sample number	Genotype	Date of birth	Date of experiment	Sex	Number of mTEChi sorted	Index1		Index2		Avr. Fragment size	nM DNA (tapestation)
1	WT	08.06.20	08.07.2020	F	7879	N702	CTAGTACG	S502	CTCTCTAT	506	60,3
2	C313X	29.07.20	03.09.2020	F	4500	N703	TTCTGCCT	S503	TATCCTCT	616	38,3
3	C313X	17.08.20	22.09.2020	F	4000	N703	TTCTGCCT	S503	TATCCTCT	593	50,3
4	Ex7Splice	20.08.20	22.09.2020	F	1500	N704	GCTCAGGA	S504	AGAGTAGA	504	25,4
5	WT1	13.08.20	22.09.2020	F	10000	N705	AGGAGTCC	S517	GCGTAAGA	575	41,6
6	C311X	13.09.20	27.10.2020	F	5500	N701	TCGCCTTA	S502	CTCTCTAT	579	71,5
7	WT2	21.09.20	27.10.2020	F	7000	N705	AGGAGTCC	S517	GCGTAAGA	540	60
8	Ex7Splice	21.09.20	27.10.2020	F	13000	N704	GCTCAGGA	S504	AGAGTAGA	512	51
9	WT	21.09.20	27.10.2020	F	8500	N701	TCGCCTTA	S502	CTCTCTAT	561	58,8
10	Ex7Splice	21.09.20	27.10.2020	F	10000	N702	CTAGTACG	S502	CTCTCTAT	540	49,5
11	Ex7Splice	15.02.21	19.03.2021	F	5000	N701	TAAGGCGA	S504	AGAGTAGA	312	73,5
12	Ex7Splice	15.02.21	19.03.2021	F	10000	N701	TAAGGCGA	S517	GCGTAAGA	258	66,7
13	WT	14.02.21	19.03.2021	M	5000	N702	CGTACTAG	S502	CTCTCTAT	268	43,9
14	C313X	14.02.21	19.03.2021	M	3500	N702	CGTACTAG	S503	TATCCTCT	190	33
15	C313X	11.02.21	19.03.2021	F	2700	N702	CGTACTAG	S517	GCGTAAGA	235	65,6
16	Ex7Splice	20.12.20	26.01.2021	F	8000	N703	AGGCAGAA	S502	CTCTCTAT	216	87,7
17	Ex7splice	20.12.20	26.01.2021	M	2700	N703	AGGCAGAA	S503	TATCCTCT	226	114
18	WT	03.01.21	09.02.2021	M	2000	N704	TCCTGAGC	S503	TATCCTCT	198	167
19	WT	03.01.21	09.02.2021	M	2300	N704	TCCTGAGC	S504	AGAGTAGA	570	14,8
20	WT	03.01.21	09.02.2021	M	2000	N706	TAGGCATG	S502	CTCTCTAT	531	16,4
21	C313X	03.01.21	09.02.2021	M	2200	N706	TAGGCATG	S504	AGAGTAGA	540	20,7
22	C313X	03.01.21	09.02.2021	F	2300	N706	TAGGCATG	S517	GCGTAAGA	241	105

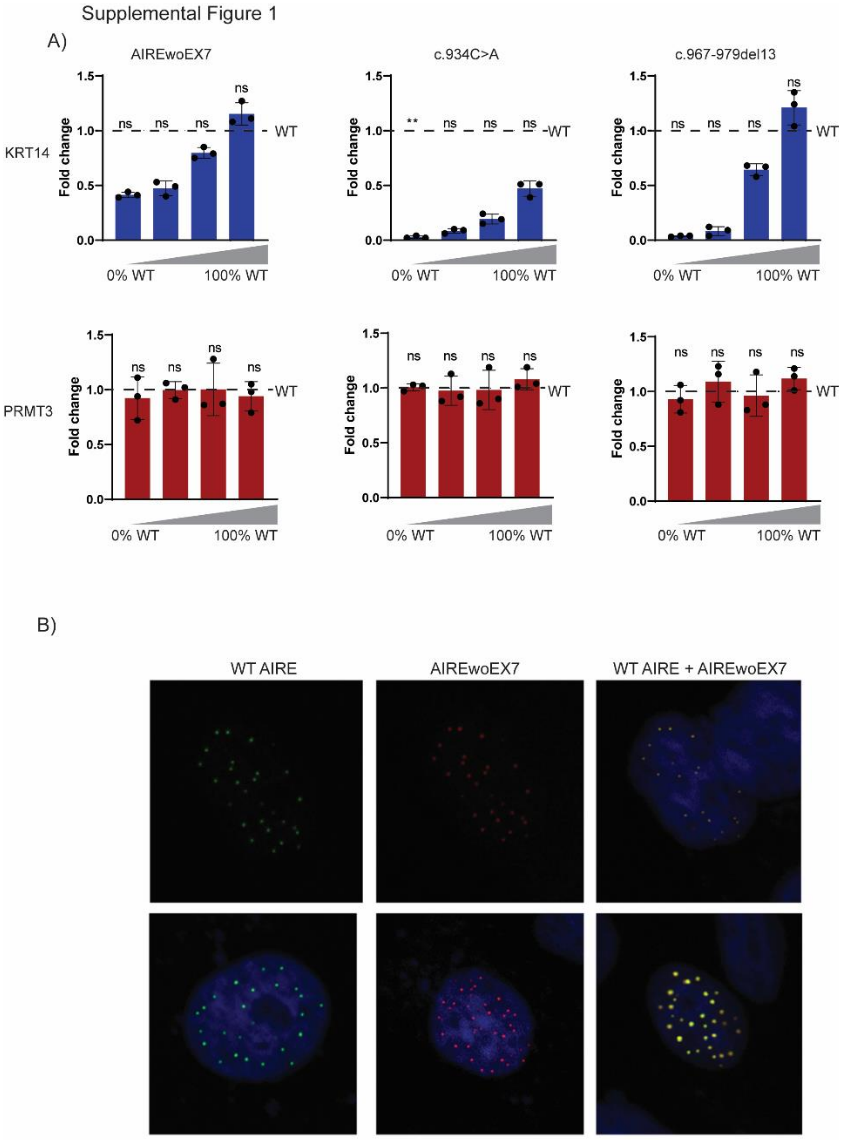

Supplemental Figure 1. Transcriptional regulation of AIRE^{-Exon7} showing a partial gene expression but no dominant negative effect.

(**A**) 4D6 cells were transfected with various amounts of WT *AIRE* and the mutants *AIRE* without exon 7 (AIRE^{-Exon7}), the c.967-979del13 mutation, and the dominant negative mutation c.934C>A (p.C311Y). The AIRE-regulated gene *keratin 14* (*KRT14*) (blue bars) and the *AIRE*-independent gene *protein arginine methyltransferase 3 (PRMT3)* (red bars) were tested using qPCR and data were normalized to *beta2-microglobulin* (*B2M*). The results are shown as fold difference (FD) relative to cells transfected with 100% WT *AIRE* (dotted line). (**B**) 4D6 cells were transfected with pCMV6 vector with AIREwoEX7 and pEGFP with WT AIRE. Staining with a phycoerytrin conjugated anti-DYDDDDK was used for the AIREwoEX7. Both WT and mutant show a similar nuclear localization forming typical speckles, and experiments with co-transfected 4D6 cells revealed co-localization of mutant and WT AIRE. Error bars (A) represents standard deviation (SD) of two to four separate experiments. *p<0.05 and **p<0.001 using t-test comparing mutant and WT AIRE.

Supplemental Figure 2

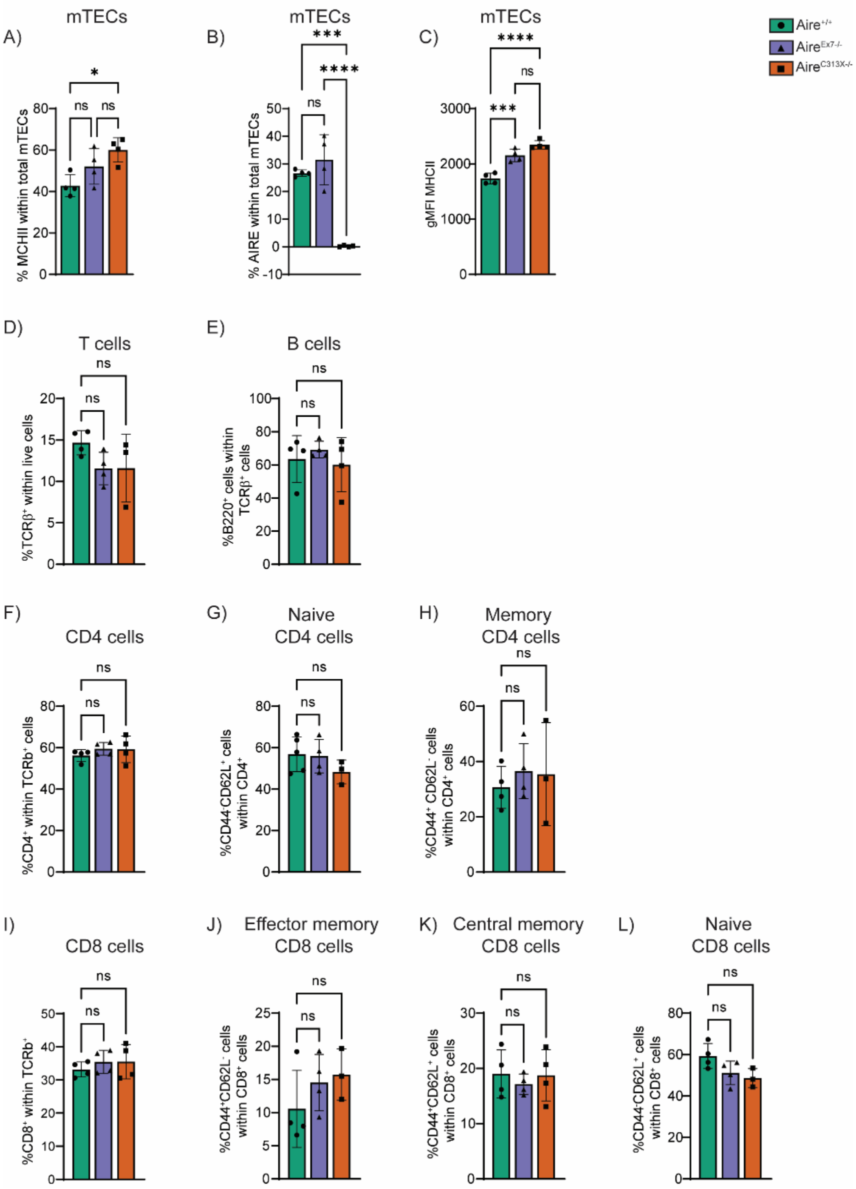

Supplemental Figure 2: Peripheral T and B cells in Aire$^{Ex7-/-}$or Aire^{C313X} mice: A) MHCII expression abd B) Aire expression within total mTECs. C) gMFI of MHCII. D) The frequencies of T cells (TCRb+ cells) and E) B cells (CD19+ cells) in the spleen of Aire$^{Ex7-/-}$or Aire^{C313X} mice were similar to Aire$^{+/+}$ littermates. F) Within the TCRb+CD4+ subset equal amounts of cells were found, together with a similar distributeion of G) naïve and H) memory CD4+ T cells in the spleen from Aire$^{Ex7-/-}$or Aire^{C313X} mice, compared to Aire$^{+/+}$ littermates. I) Within the TCRb+CD8+ compartment the frequenies of cells were similar between the Aire$^{Ex7-/-}$or Aire^{C313X} mice, compared to Aire$^{+/+}$ littermates. A similar distributeion of J) effector memory, K) central memory and L) naïve CD8+ T cells was found in the spleen from Aire$^{Ex7-/-}$or Aire^{C313X} mice, compared to Aire$^{+/+}$ mice. Error bars represents standard deviation (SD) of three to four separate experiments. *p<0.05 and **p<0.001 using t-test comparing mutant and Aire$^{+/+}$ mice.

Supplemental figure 3

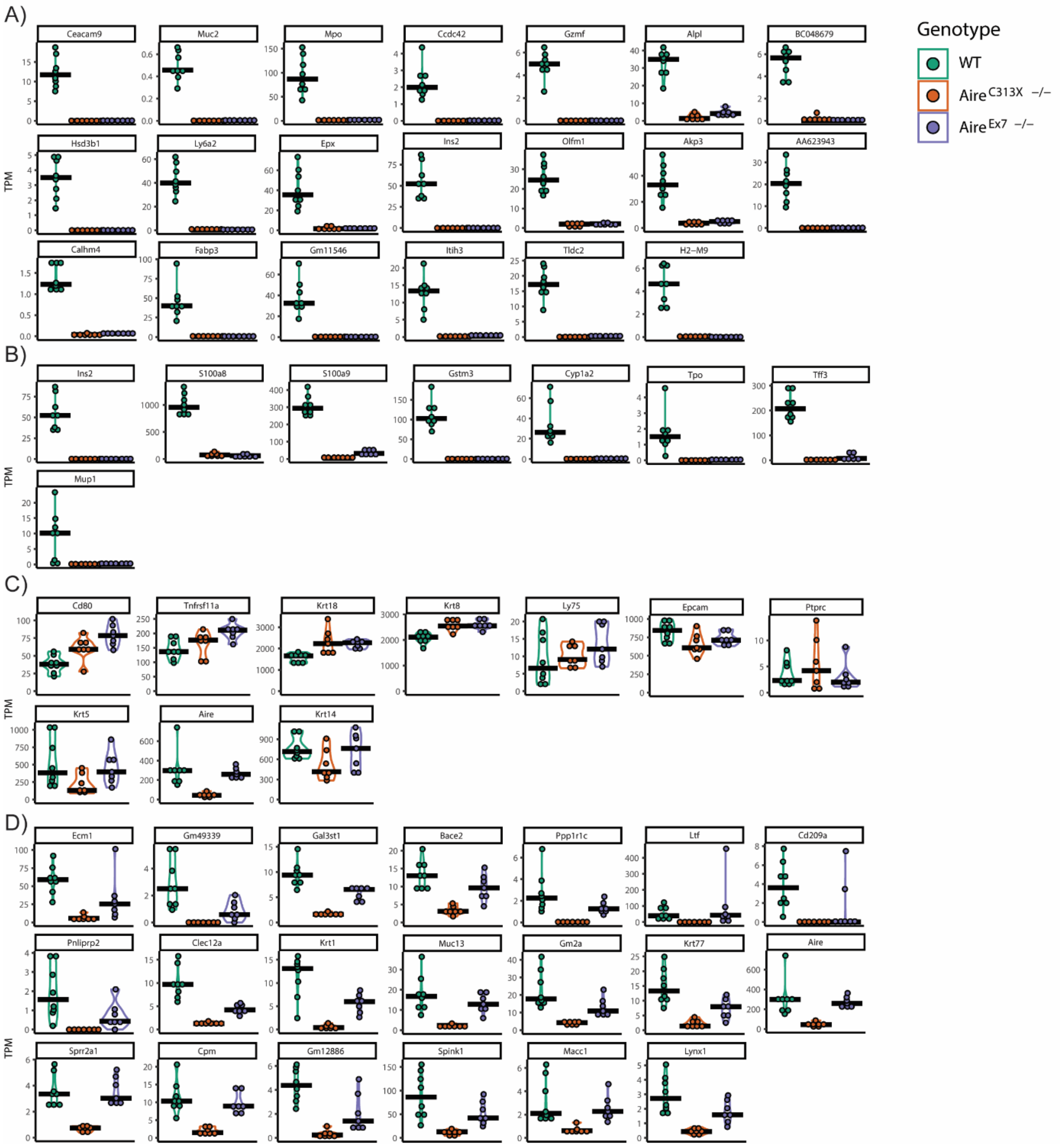

Supplemental Figure 3. Absolute expression of different subsets of differentially expressed genes. A) Violin plots of the absolute expression in transcripts per million (TPM) of the 20 most significant genes in the comparison between Aire$^{Ex7-/-}$ and WT. B) Violin plots of TPM of known Aire regulated genes. C) Violin plots of TPM of known mTEC related genes. D) Violin plots of TPM of the 20 most significant genes that are less expressed in the comparison between Aire^{C313X} and WT that are also significant in the comparison between Aire$^{Ex7-/-}$ and Aire^{C313X}. Black bars denote median tpm within group.

Supplemental figure 4

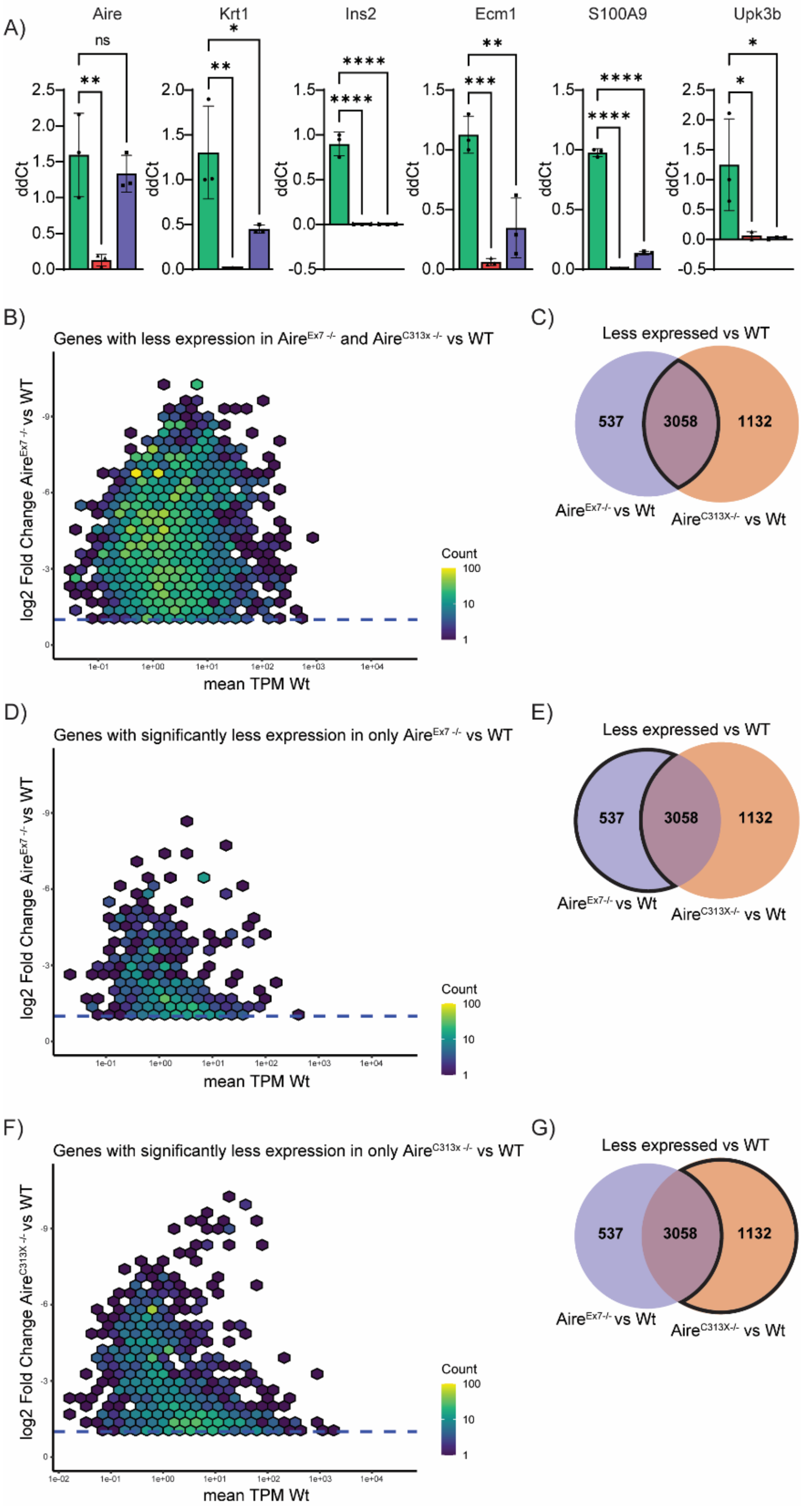

Supplemental Figure 4. Characterization of uniquely downregulated genes. Hexbin density plot of absolute and relative expression values in downregulated genes plotted with transcripts per million in WT mice on the x axis and Log2FoldChange on the y axis and Venn diagram highlighting the specific subset of significantly differentially expressed genes. Hexes colored by the number of between 1-100 genes found within the area of the hex. (**A**) all significantly downregulated genes in the comparison of both mutant mice populations ($Aire^{Mut}$) compared to wildtype. (**B**) Genes significantly downregulated in $Aire^{C313X-/-}$ mice compared to wildtype mice, but not significantly downregulated in $Aire^{Ex7-/-}$ mice or when looking at both mutant mice compared to wildtype. (**C**) Genes significantly downregulated in $Aire^{Ex7-/-}$ mice compared to wildtype mice, but not significantly downregulated $Aire^{C313X-/-}$ mice or when looking at both mutant mice compared to WT. Error bars (A) represents standard deviation (SD) of three separate experiments. *p<0.05 and **p<0.001 using t-test comparing mutant and $Aire^{+/+}$ mice.

Supplemental Figure 5

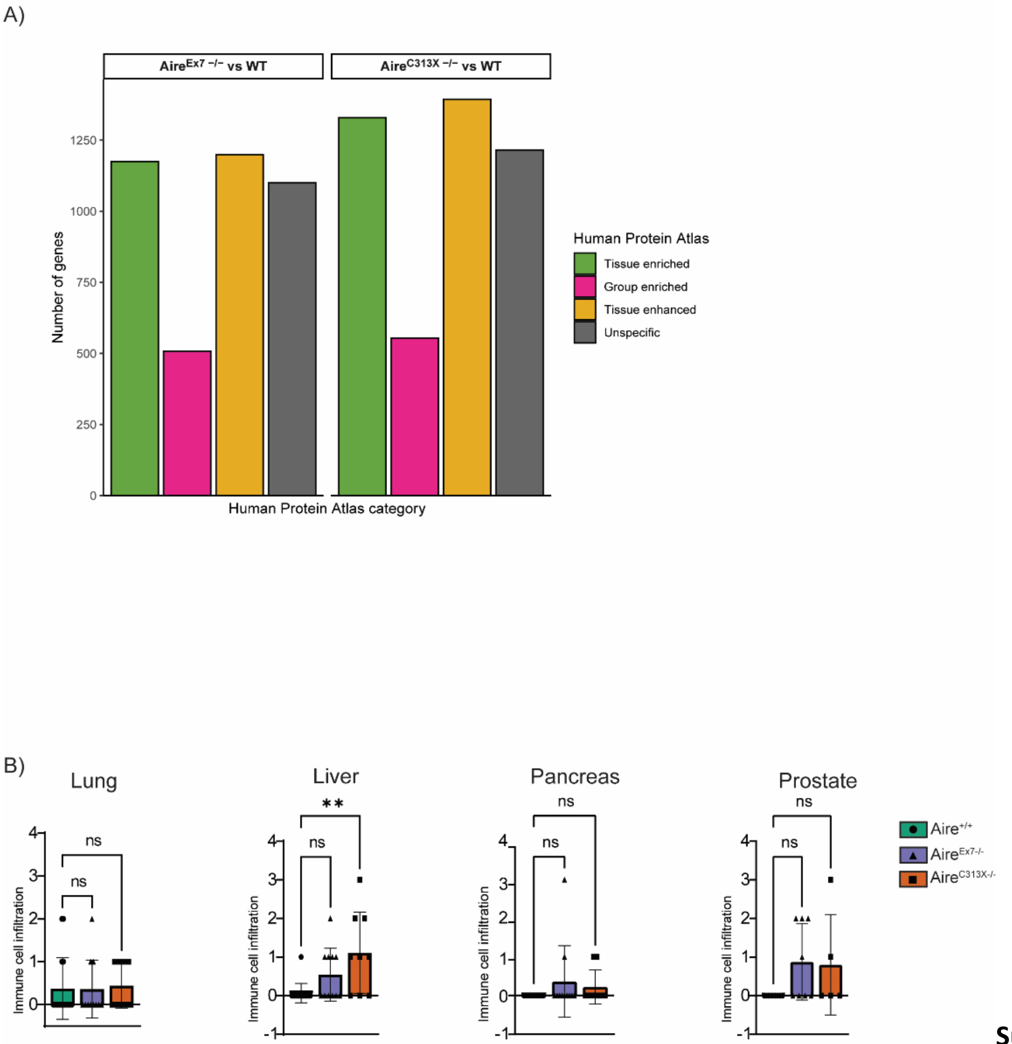

Supplemental Figure 5: Humanised gene expression in mTECs and infiltration of immunecells in different tissues. (A) The significantly downregulated genes from the mTEC analysis were humanized with biomart and examined against the Human Protein Atlas data on tissue specific genes. Tissue enriched: At least four-fold higher mRNA level in a particular tissue compared to any other tissue. Group enriched: At least four-fold higher average mRNA level in a group of 2-5 tissues compared to any other tissue. Tissue enhanced: At least four-fold higher mRNA level in a particular tissue compared to the average level in all other tissues. (B) Infiltration of immune cells were evident in liver tissue, most prominent in the Aire$^{C313X-/-}$ mice, here investigating lung, liver, pancreas, and prostate. Error bars represents mean ± SD. *p<0.05 and **p<0.001 using one-way Anova with multiple comparison, comparing Aire$^{C313X-/-}$ and Aire$^{Ex7-/-}$ to Aire$^{+/+}$.

Supplemental Figure 6

A)

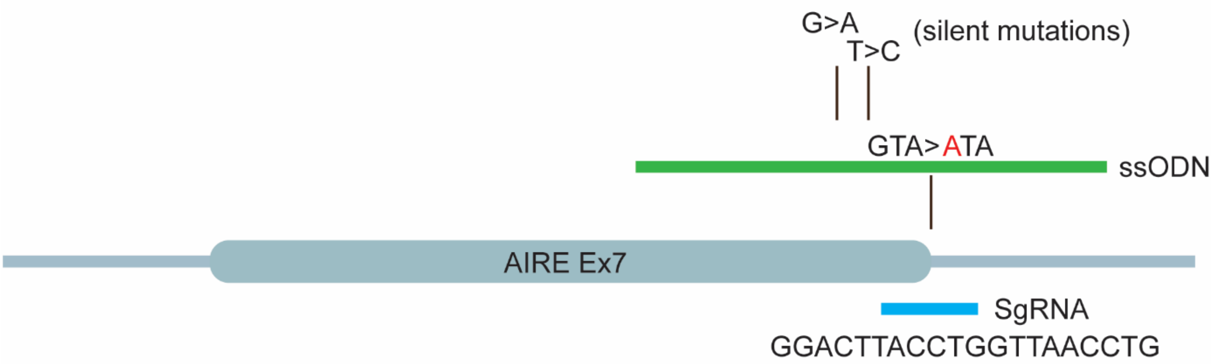

Supplemental Figure 6: Introducing the splice mutation by Crispr/Cas9 homology directed repair. The single guide RNA (sgRNA) and single-stranded oligodeoxynucleotide (ssODN) are shown, introducing the desired splice mutation.

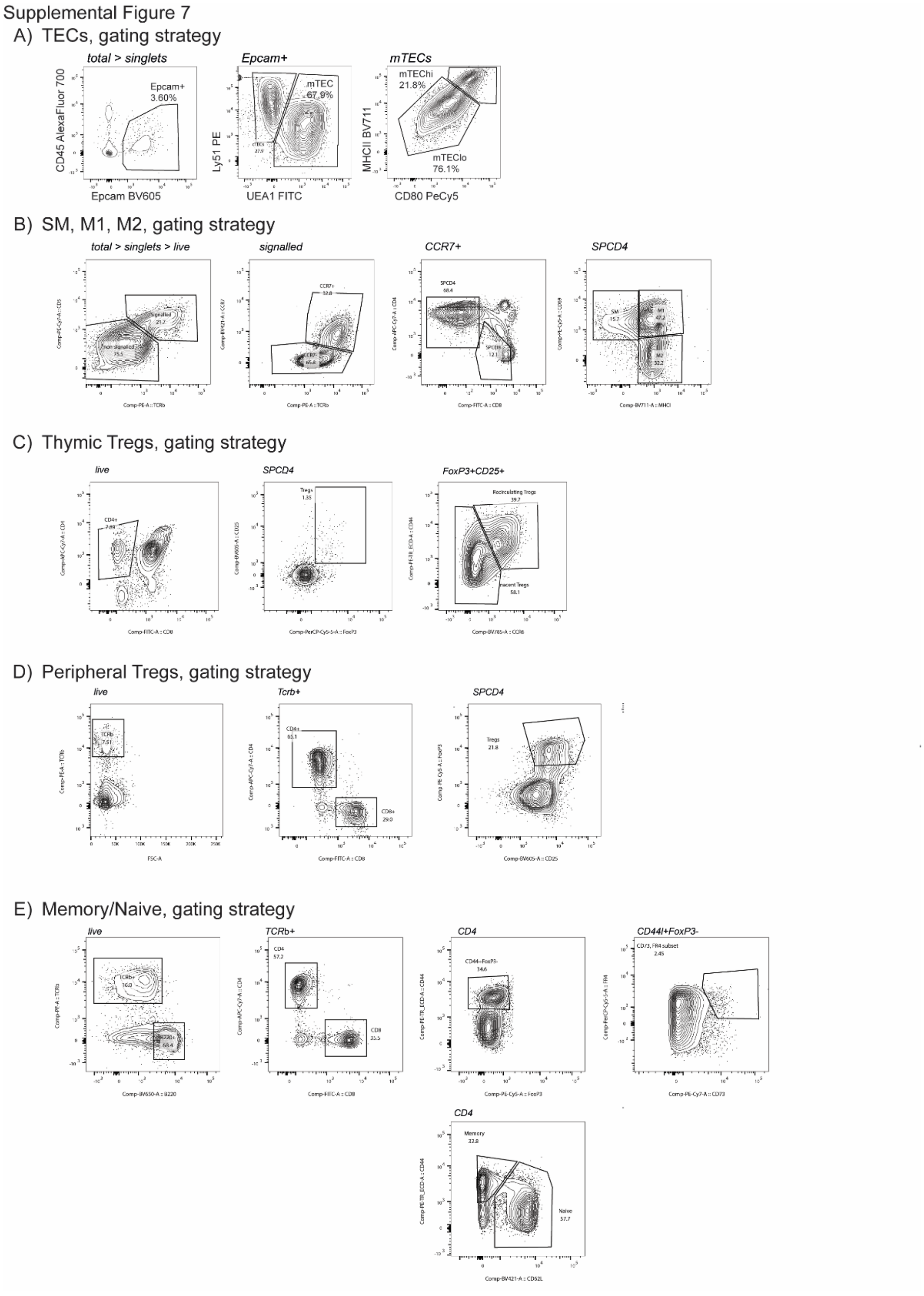

Supplemental Figure 6: Representative gating strategy. Representative gating for identification of **A)** cTEC, mTEC^{hi} and mTEC^{lo}, **B**) Semimature (SM), mature1 (M1) and mature 2 (M2) thymocytes, **C**) total thymic Tregs (CD4+CD25+FoxP3+), nascent Tregs (CD4+CD25+FoxP3+CCR6-), and recirculating Tregs (CD4+CD25+FoxP3+CD44hiCCR6+), **D**) peripheral Tregs and E) memory/naïve T cells by Flow Cytometry.
